# Bioinformatics analysis of myelin-microbe interactions suggests multiple types of molecular mimicry in the pathogenesis of multiple sclerosis

**DOI:** 10.1371/journal.pone.0308817

**Published:** 2024-12-30

**Authors:** Ali Bigdeli, Mostafa Ghaderi-Zefrehei, Bluma J. Lesch, Mehrdad Behmanesh, S. Shahriar Arab

**Affiliations:** 1 Department of Biophysics, School of Biological Sciences, Tarbiat Modares University, Tehran, Iran; 2 Department of Animal Genetics, Faculty of Agriculture, Yasouj University, Yasouj, Iran; 3 Department of Genetics, Department of Obstetrics, Gynecology & Reproductive Sciences, and Yale Cancer Center, Yale School of Medicine, New Haven, CT, United States of America; 4 Department of Molecular Genetics, School of Biological Sciences, Tarbiat Modares University, Tehran, Iran; University of Jeddah, SAUDI ARABIA

## Abstract

Multiple sclerosis (MS) is a devastating autoimmune disease that leads to the destruction of the myelin sheath in the human central nervous system (CNS). Infection by viruses and bacteria has been found to be strongly associated with the onset of MS or its severity. We postulated that the immune system’s attack on the myelin sheath could be triggered by viruses and bacteria antigens that resemble myelin sheath components. An in-silico bioinformatics approach was undertaken in order to identify viral and bacterial antigens that resemble myelin oligodendrocyte glycoprotein (MOG) and myelin basic protein (MBP). To this end, we simultaneously analyzed both protein structures and amino acid sequences from viral and bacterial proteins and compared them to MOG and MBP. Possible associations between MBP and human parvovirus B19 (HPV-B19) and adeno-associated virus 4 (AAV-4) capsid protein structures were identified. MBP and MOG were associated with antigens from different viruses and bacteria, including *Aspergillus species*, *Lactobacillus*, *Burkholderia*, *Clostridium*, *Schizosaccharomyces*, SARS-CoV-2, and some gut flora metabolites. We also identified similarities between MBP and MOG proteins and bile salt hydrolase (BSH), glycosyltransferase (WcfQ), and Wzy enzymes. Identical amino acids between MBP and BSH at the active site, and protected amino acids in MOG aligning with WcfQ and Wzy enzymes were observed. Overall, our results offer valuable insights into the role of different viral and bacterial protein antigens in MS pathogenesis and suggest the possibility of identifying new therapeutic targets using in silico bioinformatics approaches. Our proposed approach could also likely be adapted for other CNS diseases in order to develop new biological insights and treatments.

## Introduction

Multiple sclerosis (MS) is a central nervous system (CNS) disease that produces inflammatory plaques in the brain, spinal cord, and optic nerve, with associated nerve cell degeneration [1, 2]. The underlying cause of MS is autoimmune attack on myelin, the insulating layer that allows nerves to send electrical impulses over long distances. Myelin is made up of specialized glial cell membranes that wrap multiple times around a neuron’s axon sheath and contain specialized proteins and lipids [3]. Some viruses including Epstein-Barr virus (EBV), human herpesvirus 6 (HHV-6), and the human endogenous retroviruses (HERV) family, as well as bacteria like *Helicobacter pylori*, *Chlamydia pneumonia*, and *Staphylococcus aureus* enterotoxins, are associated with the development or exacerbation of MS [4]. This association has been postulated to be based on molecular mimicry by the viral or bacterial proteins, a process by which similar antigenic compounds generated by microorganisms cause autoimmune diseases by mimicking endogenous proteins and triggering an immune response [5, 6]. Molecular mimicry by pathogens has attracted significant attention to finding interactions between infectious agents and MS [7–10]. Two myelin proteins thought to be targets for a molecular mimicry mechanisms are myelin basic protein (MBP) and myelin oligodendrocyte glycoprotein (MOG) [10, 11].

Numerous studies have investigated the immune response in MS patients, revealing that the presence of peptides from two proteins—one from Epstein-Barr Virus (EBV) and the other from the bacterium *Mycobacterium paratuberculosis* (MAP)—in the cerebrospinal fluid and serum trigger autoimmunity [12]. EBV, in particular, has been strongly associated with the onset of MS [13]. The interferon regulatory factor 5 protein (IRF-5) epitope matches EBV and MAP antigens and elicits a humoral immune response in MS patients [14]. Additional peptides derived from EBV and MAP that cross-react with myelin proteins have been discovered through the use of myelin-targeting antibodies and are thought to be involved in the progression of MS [15, 16]. In addition to EBV, other viral pathogens including Parvovirus B19 species (*Parvoviridae* virus family, HPV-B19) have been linked to both autoimmune disorders and CNS diseases, including MS [17–21]. HPV-B19 shares a secondary and tertiary structure with serotype 4 of an adeno-associated virus (AAV), also from the *Parvoviridae* family. There is active research into the structure and function of AAV capsid proteins with the goal of developing approaches to modulate their immune responses [22–24]. Additionally, there are several viruses in the *herpesviridae* family, including human herpesvirus 6 (HHV-6), which have been implicated in the development of autoimmune disorders including MS [25, 26].

In bacteria, the epsilon toxin of the pathogen *Clostridium perfringens* is known to cause brain damage in both animals and humans by affecting their neurological systems [27]. The presence of antibodies to epsilon toxin in some MS patients has been reported [28], suggesting that *C*. *perfringens* epsilon toxin could also play a role in the development of MS in some individuals. In addition, synergy between native gut bacteria *Lactobacillus reuteri* and OTU0002 *Erysipelotrichaceae* in the mouse can lead to intestinal T-cell responses to MOG in the CNS, potentially resulting in autoimmune encephalitis [29]. Part of the MOG and MBP protein sequences were also found to be similar to enzyme sequences from gut bacteria involved in butyric acid and polysaccharide A metabolism, including bile salt hydrolase (BSH) [30, 31].

Our aim in this study was to investigate the structural and sequence relationships between microorganism-derived proteins and myelin-based proteins. We evaluated structural relationships and amino acid sequence similarity between proteins from the viral and bacterial microorganisms discussed above and MOG and MBP by means of in-silico bioinformatics. In the first step, we focused on the 3D structure, conserved regions, and sequence association of MBP with viral capsid protein structures of HPV B19 and AAV-4. We then reviewed previous studies to identify parts of the myelin protein amino acid sequences that contribute to cases of molecular mimicry between microorganism proteins and MOG and MBP, and found additional pathogenic microorganisms where structural proteins contained matched peptide sequences, suggesting that these pathogens could also trigger autoimmunity. Finally, we also identified similar peptide sequences in proteins from native gut microbiota and metabolites, where mimicry could cause dysbiosis in the intestine and induce an immune system response. Together, we identified potential associations with specific microorganisms and antigens that could lead to the development of new strategies for treating and preventing autoimmune disorders, particularly MS.

## Materials and methods

**Fig 1:**
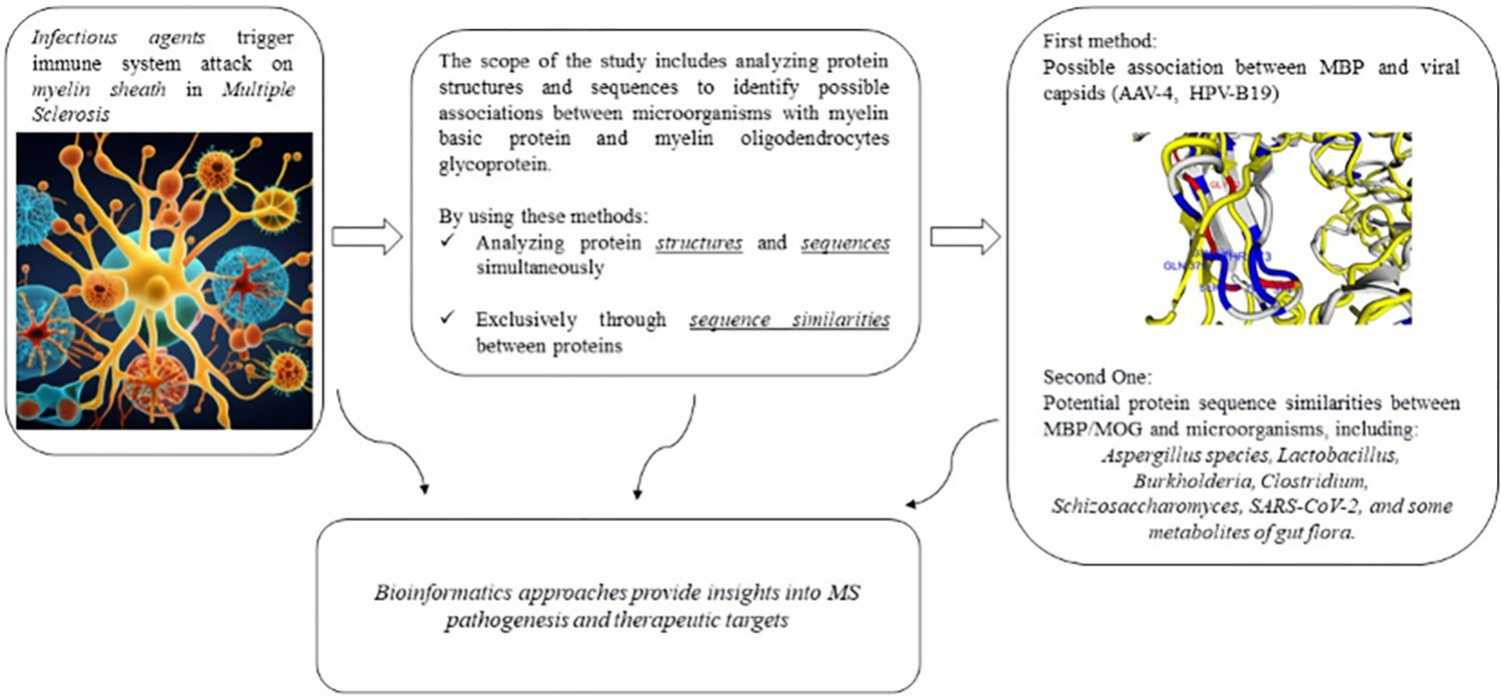
Study pipeline overview.

Fig 1 shows a comprehensive overview of the study methodology.

### Extraction of data related to MBP

**Fig 2:**
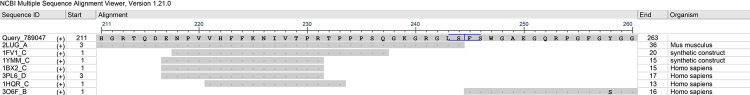
The current PDB structures related to a portion of the MBP sequence.

The amino acid sequence of MBP (accession number P02686) was obtained from UniProt (https://www.uniprot.org/). The sequence was checked in the Research Collaboratory for Structural Bioinformatics (RCSB) [32] database for the presence of a tertiary structure. Examination of the Basic Local Alignment Search Tool (BLAST) [33] and Protein Data Bank (PDB) [34] databases revealed that there are only a few known 3D structures within the 304 amino acids of the full-length protein (Fig 2).

The tertiary structures of MBP were obtained from AlphaFold (https://alphafold.ebi.ac.uk/), which uses a deep learning approach that leverages physical, biological, and bioinformatics information about protein structure and multiple sequence alignments [35, 36].

### MBP alignment with HPV-B19 and AAV-4

The HPV-B19 and AAV-4 viral capsid protein sequences were obtained from UniProt (accession number Q9PZT0 and O41855, respectively) and aligned separately with MBP using the PRALINE server [37, 38] which takes into account the predicted secondary structure during the alignment of amino acid sequences. The results of this alignment were used for further investigation of the tertiary structures. To evaluate the obtained scores and classify the amino acid alignments information, the position of each amino acid in the 3D structure was analyzed using the YASARA software [39]. The software’s structural overlap feature was utilized to analyze similarities between the two-3D viral capsid protein structures using pairwise alignment from the MUSTANG algorithm [40].

### Extraction of data related to MOG

Data on MOG were retrieved from the UniProt database using the human and mouse codes Q16653 and Q61885, respectively. In mice, the *YRSPFSRVV* motif is linked to the development of autoimmune encephalitis of the CNS and, as a result, MS [29]. This MOG motif in mice has a specified tertiary structure that was discovered using X-ray crystallography [41]. The equivalent motif in human is *YRPPFSRVV*, and the tertiary structure of this human domain is also included in the PDB database.

### Predicting antibody epitope

In order to predict antigenic regions in each of the proteins studied, we tested three online tools: Bepipred Linear Epitope Prediction 2.0 (BepiPred-2.0) (Sequential B-Cell Epitope Predictor) [42], Bcepred (http://crdd.osdd.net/raghava/bcepred/) [43] and the Kolaskar & Tongaonkar antigenicity scale [44]. However, we observed discrepancies in the results obtained from each software program. To ensure consistency and avoid confusion, we decided to report data from only one software program. We selected the BepiPred-2.0 method as the preferred tool for our analysis, based on the superior performance of BepiPred-2.0 compared to other available tools for predicting B-cell epitopes.

### Microbe motifs and MS

The information used as input in the alignments and adaptations of this study was carefully selected to ensure accuracy and reliability. Table 1 displays the data gathered from the investigations carried out in [12, 15].

**Table 1:** The sequence information for *Mycolicibacterium paratuberculosis*, Epstein-Barr virus, and *Homo sapiens*, along with the corresponding protein names for each sequence (derived from previous studies) [12, 15].

*Organism*	*Sequence information*			*Protein name*	*reference*
*Mycolicibacterium paratuberculosis* (strain ATCC BAA-968 / K-10)	121	*PGRRPFTRKELQ*	132	Tyr recombinase domain-containing protein (MAP_0106)	[12]
Epstein-Barr virus (strain B95-8) (HHV-4)	400	*PGRRPFFHPVGEAD*	413	Epstein-Barr nuclear antigen 1 (EBNA1)	
*Homo sapiens* (Human)	85	*PGSRPHLIRLFSRD*	98	MBP (MBP_HUMAN)	
*Mycolicibacterium paratuberculosis* (strain ATCC BAA-968 / K-10)	18	*AVVPVLAYAAARLLL*	32	Uncharacterized protein (MAP_4027)	[15]
Epstein-Barr virus (strain B95-8) (HHV-4)	305	*AVPVLAFDAARLRLLE*	320	Inner tegument protein (BOLF1)	
*Homo sapiens* (Human)	424	*RLLLEMFSGEL*	434	Interferon regulatory factor 5 (IRF5_HUMAN)	

Table 2 presents the sequence similarity information between *Lactobacillus and OUT002* proteins and MOG.

**Table 2:** Protein sequence similarity and conserved amino acids, with bolded residues such as Arginine, Phenylalanine, Arginine, and Valine (observed in the cited study) [29].

*Organism*	*Sequence information*			*Protein name*
*Lactobacillus reuteri*	172	*K* ***R*** *EG* ***F*** *V* ***RV*** *Q*	180	UvrABC system protein A
*Lactobacillus reuteri* (strain DSM 20016)	324	*G* ***R*** *LT* ***F*** *L* ***RV*** *Y*	332	Elongation factor G
*Lactobacillus reuteri* (strain DSM 20016)	122	*K* ***R*** *IA* ***F*** *S* ***RV*** *Y*	130	Amino acid ABC transporter substrate-binding protein
OTU0002	N. A	*E* ***R*** *DG* ***F*** *T* ***RV*** *L*	N. A	Xaa-Pro Aminopeptidase
*Erysipelotrichaceae*				
*Mus musculus*	68	*Y* ***R*** *SP* ***F*** *S* ***RV*** *V*	76	MOG (MOG_MOUSE)

Table 3 summarizes the other key information gathered from previous research that served as a reference for the present study, including details on the proteins and pathogenic motifs investigated in this study. PRALINE server was used for finding the sequence alignments [37, 38]. The amino acid alignment score ranges from 0 to 10, where 0 indicates no similarity and a star (10) indicates identity.

**Table 3:** A summary of the data gathered from previous researches.

*Protein*	*Sequence*	*Organism*	*References*
U24 protein	*MDRPRTPPPSYSE*	Human herpesvirus 6B (strain Z29) (HHV-6 variant B) (Human B lymphotropic virus)	[45]
Elongation factor G	*GRLTFLRVY*	*Lactobacillus reuteri* (strain DSM 20016)	[29]
UvrABC system protein A	*KREGFVRVQ*	*Lactobacillus reuteri*	
Aminopeptidase (AP)	*ERDGFTRVL*	*Erysipelotrichaceae bacterium* OTU0002	
Amino acid ABC transporter substrate-binding protein	*KRIAFSRVY*	*Lactobacillus reuteri*	
Tyr recombinase domain-containing protein	*PGRRPFTRKELQ*	*Mycolicibacterium paratuberculosis* (strain ATCC BAA-968 / K-10) (Mycobacterium paratuberculosis)	[12, 15]
Epstein-Barr nuclear antigen 1	*PGRRPFFHPVGEAD*	Epstein-Barr virus (strain B95-8) (HHV-4) (human herpesvirus 4)	
Inner tegument protein	*AVPVLAFDAARLRLLE*	Epstein-Barr virus (strain B95-8) (HHV-4) (human herpesvirus 4)	
Uncharacterized protein (MAP_4027)	*AVVPVLAYAAARLLL*	*Mycolicibacterium paratuberculosis* (strain ATCC BAA-968 / K-10) (*Mycobacterium paratuberculosis*)	
Epsilon-toxin type B	*TGVSLTTSYSFANTN*	*Clostridium perfringens*	[27]
Conjugated bile acid hydrolase	*All of Sequence*	*Clostridium perfringens* (strain 13 / Type A)	[31]
Conjugated bile acid hydrolase	*All of Sequence*	*Lactobacillus plantarum* (strain ATCC BAA-793 / NCIMB 8826 / WCFS1)	
Capsular polysaccharide biosynthesis protein	*All of Sequence*	*Bacteroides fragilis* (strain ATCC 25285 / DSM 2151 / JCM 11019 / NCTC 9343)	[30]

**Fig 3:**
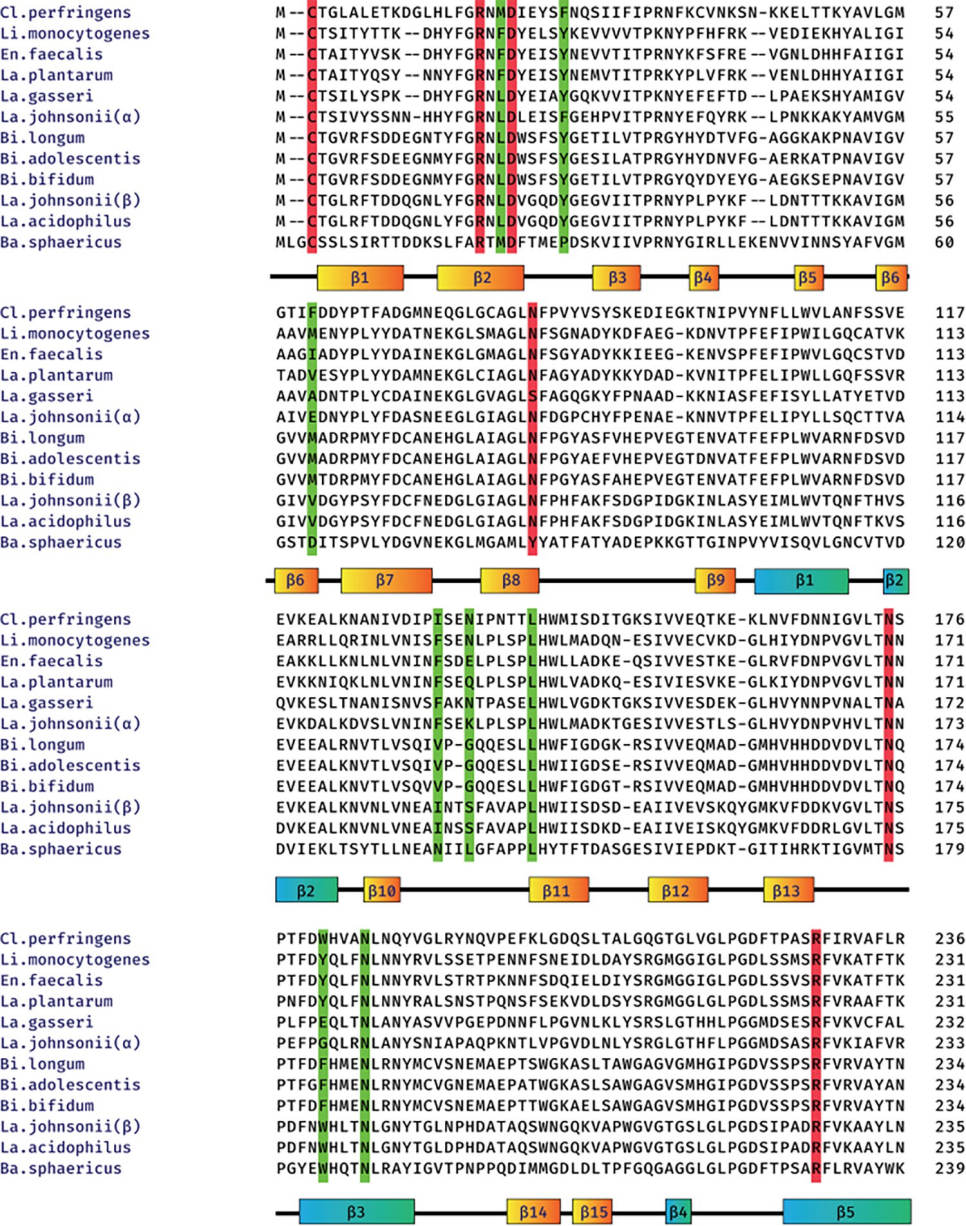
Alignment of BSH amino acid sequence from the bacteria and associated enzymes that participate in the butyric acid metabolic pathway. The active site’s amino acids are highlighted in red, based on the structure of the BSH from *C*. *perfringens*.

**Fig 4:**
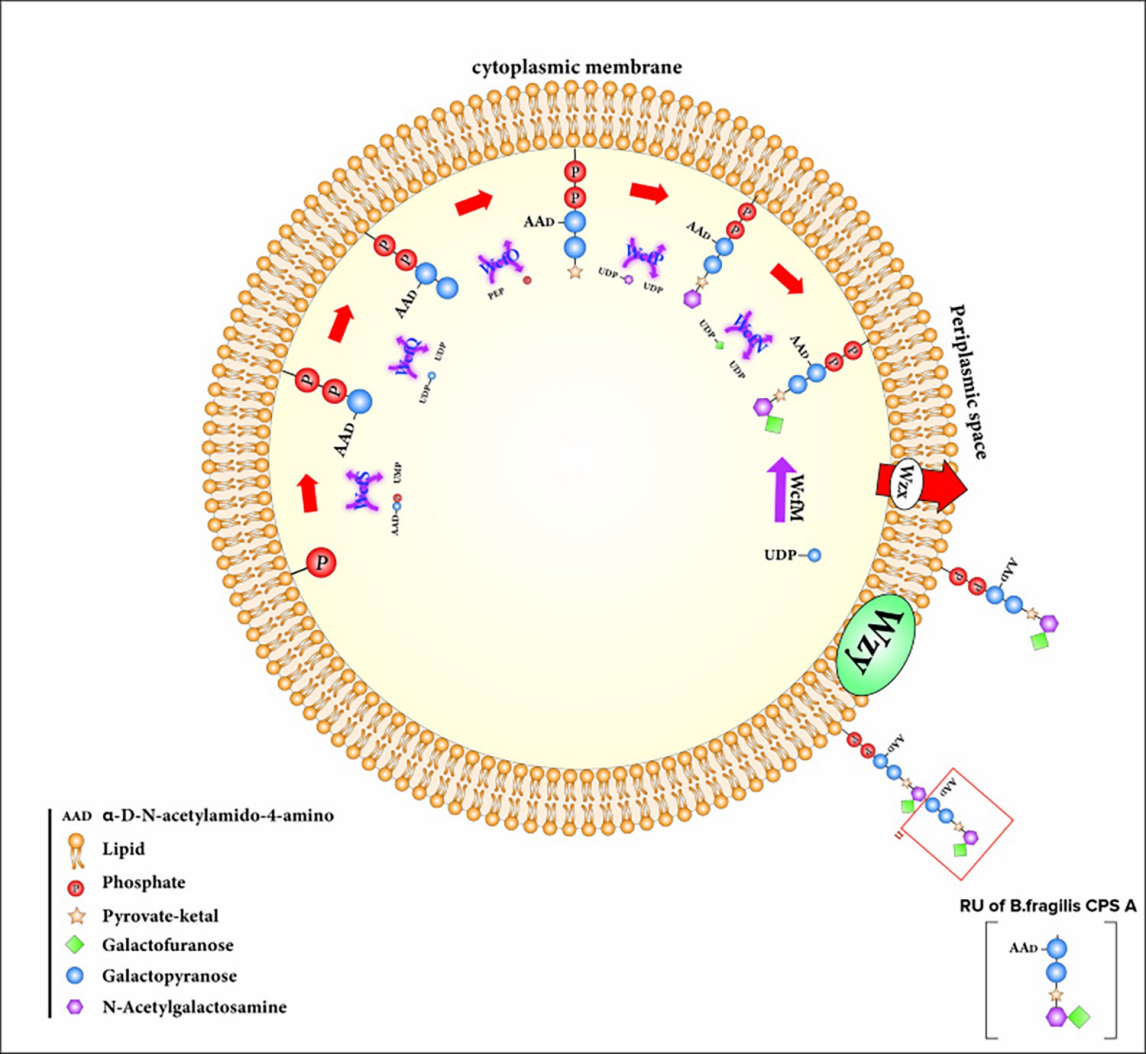
The complex metabolic pathway involved in the production of polysaccharide A, a molecule with potential neuroprotective effects.

Based on [30, 31], Fig 3 shows an alignment of BSH protein sequences from various bacteria, while Fig 4 provides a visual representation of the various enzymes involved in the synthesis of the polysaccharide A.

## Results

### Structural similarity analysis

#### HPV-B19 capsid protein

**Fig 5:**
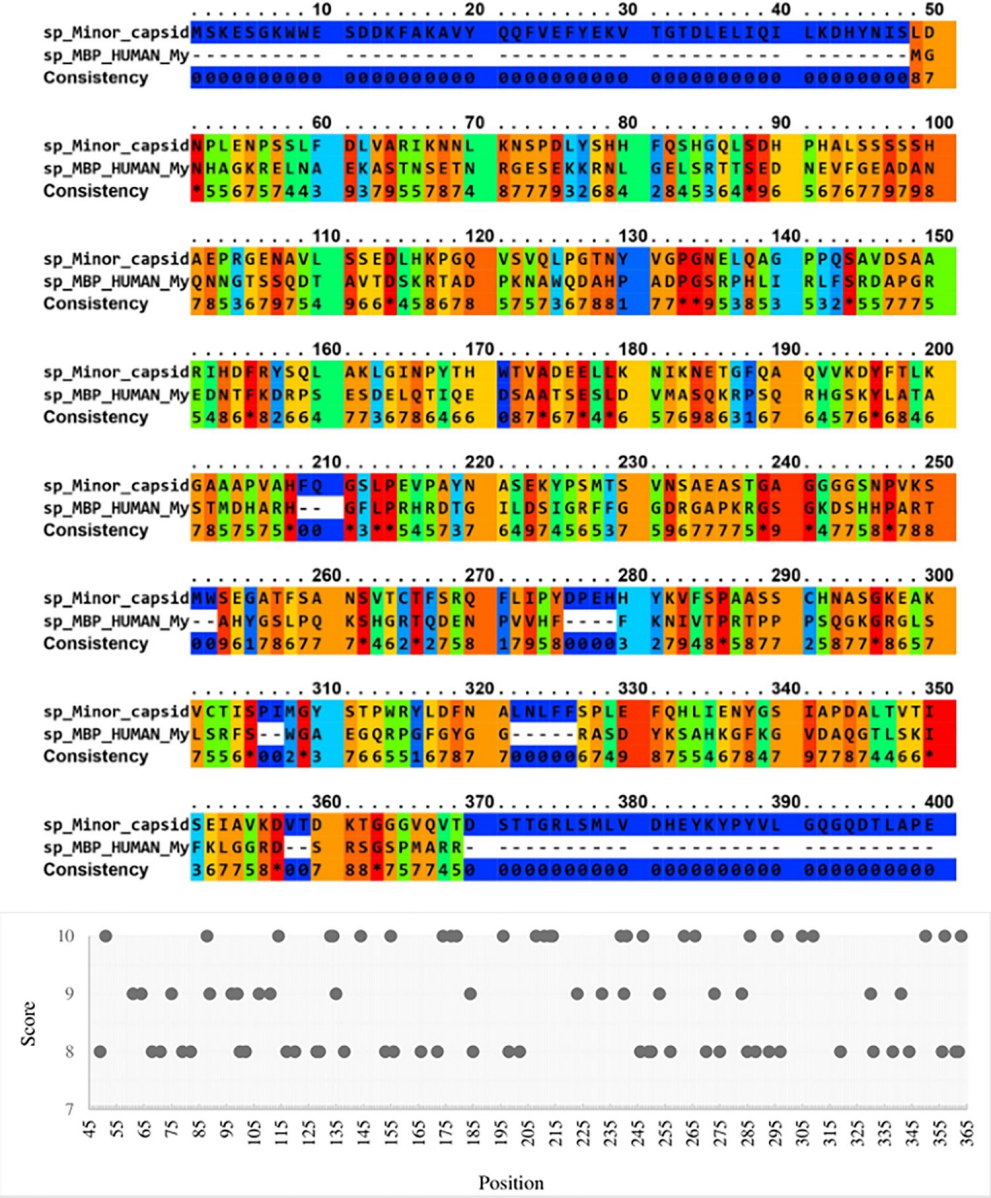
**a)** Amino acid sequence alignment of MBP with the HPV-B19 viral capsid protein. The first line displays the viral capsid amino acid sequence, the second line displays the MBP amino acid sequence, and the third line displays the scores associated with the amino acid alignment between the two sequences. In the amino acid alignment, the colors blue and red represent the gap and identity mode, respectively. Stars represent identity between all aligned amino acids. **b)** Position-specific scoring matrix for amino acid sequence alignment between HPV-B19 viral capsid protein and MBP protein.

We used the protein sequence (UniProt accession number Q9PZT0) and tertiary structure (RCSB database accession number 1S58, obtained by X-ray diffraction) of the HPV-B19 capsid protein to perform a structure and sequence alignment with MBP. The sequence alignment of the two proteins is displayed in Fig 5A. The horizontal axis in Fig 5B shows the alignment position, and the vertical axis shows the scoring above eight on alignment. An alignment score of 8.0 or higher is unlikely to occur by random chance, thus suggesting potential functional or structural relevance. Interestingly, Serine and Lysine amino acids were most frequently observed to have an alignment score above 8 in this evaluation.

#### AAV-4 capsid protein

**Fig 6:**
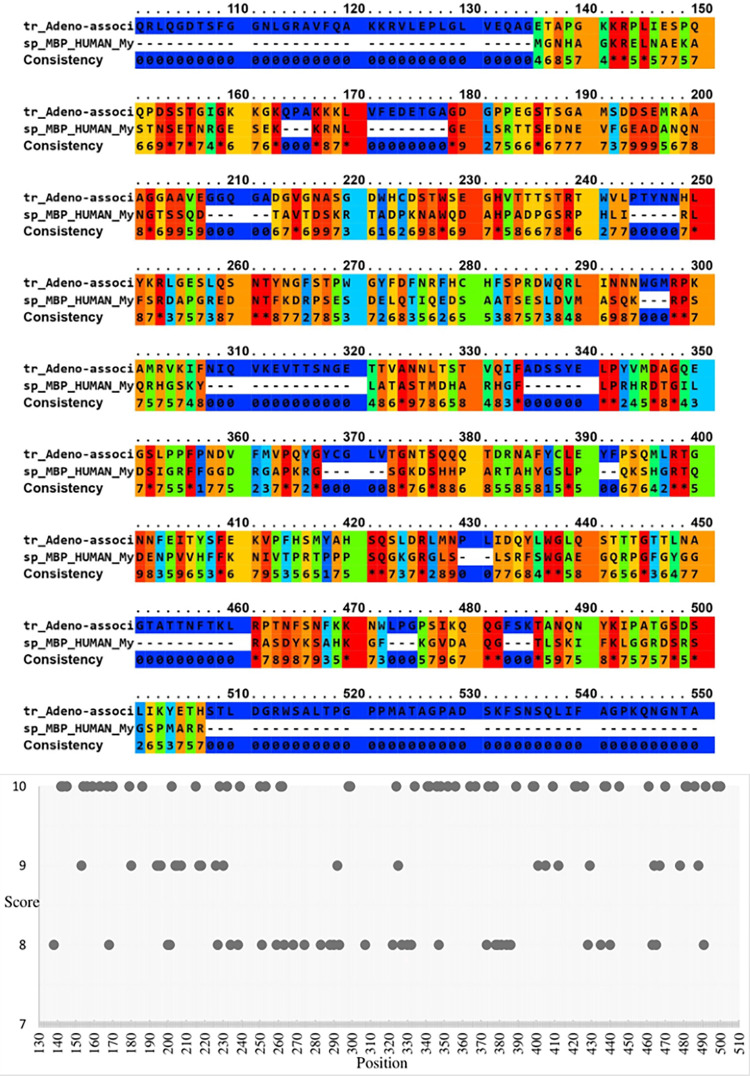
**a)** Alignment of AAV-4 viral capsid protein with MBP amino acids sequence. The first line contains the amino acid sequence of the viral capsid coat, while the second line contains the amino acid sequence of the MBP. The third line depicts the consistency of each amino acid alignment from 0 to 10 (star). The similarity and identity of two amino acids are represented by points, with 0 representing the gap mode and a star indicating identity between all aligned amino acids. **b)** Scoring matrix for aligning amino acid sequences of AAV-4 viral capsid protein and MBP protein.

We used the protein sequence (UniProt accession number O41855) and tertiary structure (RCSB database accession number 2G8G, obtained by X-ray diffraction) of the AAV-4 capsid protein to perform a structure and sequence alignment with MBP. We identified AAV-4 for this analysis because the tertiary structure of HPV-B19 and AAV-4 capsid proteins have a root-mean-square deviation (RMSD) of 9.64 for the Cα atoms, consistent with divergence of the protein sequence overall but high similarity in at least some regions. The alignment result of protein sequences is shown in Fig 6A. In Fig 6B, the alignment diagram of section A is drawn, where the horizontal axis shows the alignment position, and the vertical axis shows scoring above eight on alignment. It is noteworthy that the amino acids threonine, asparagine, and glutamine most frequently have high alignment scores in this analysis.

#### Tertiary structure alignments

**Fig 7:**
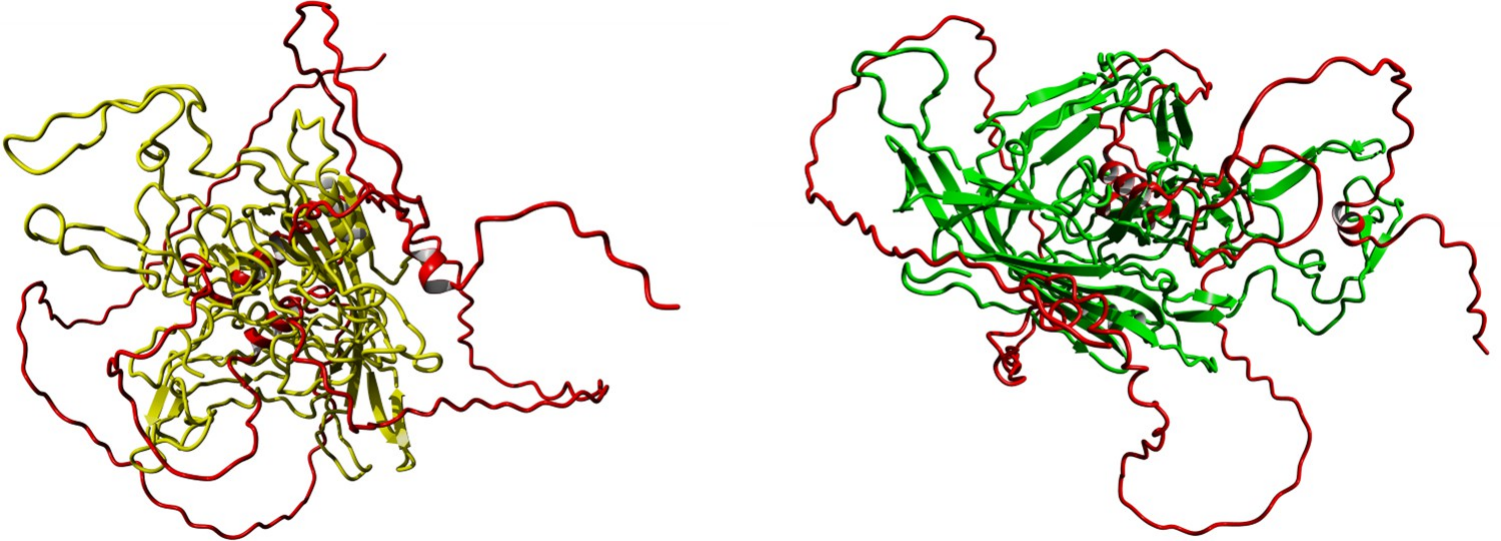
**a)** Alignment of tertiary structure between HPV-B19 capsid protein and MBP. **b)** Alignment of tertiary structure between AAV-4 capsid protein and MBP.

**Fig 8:**
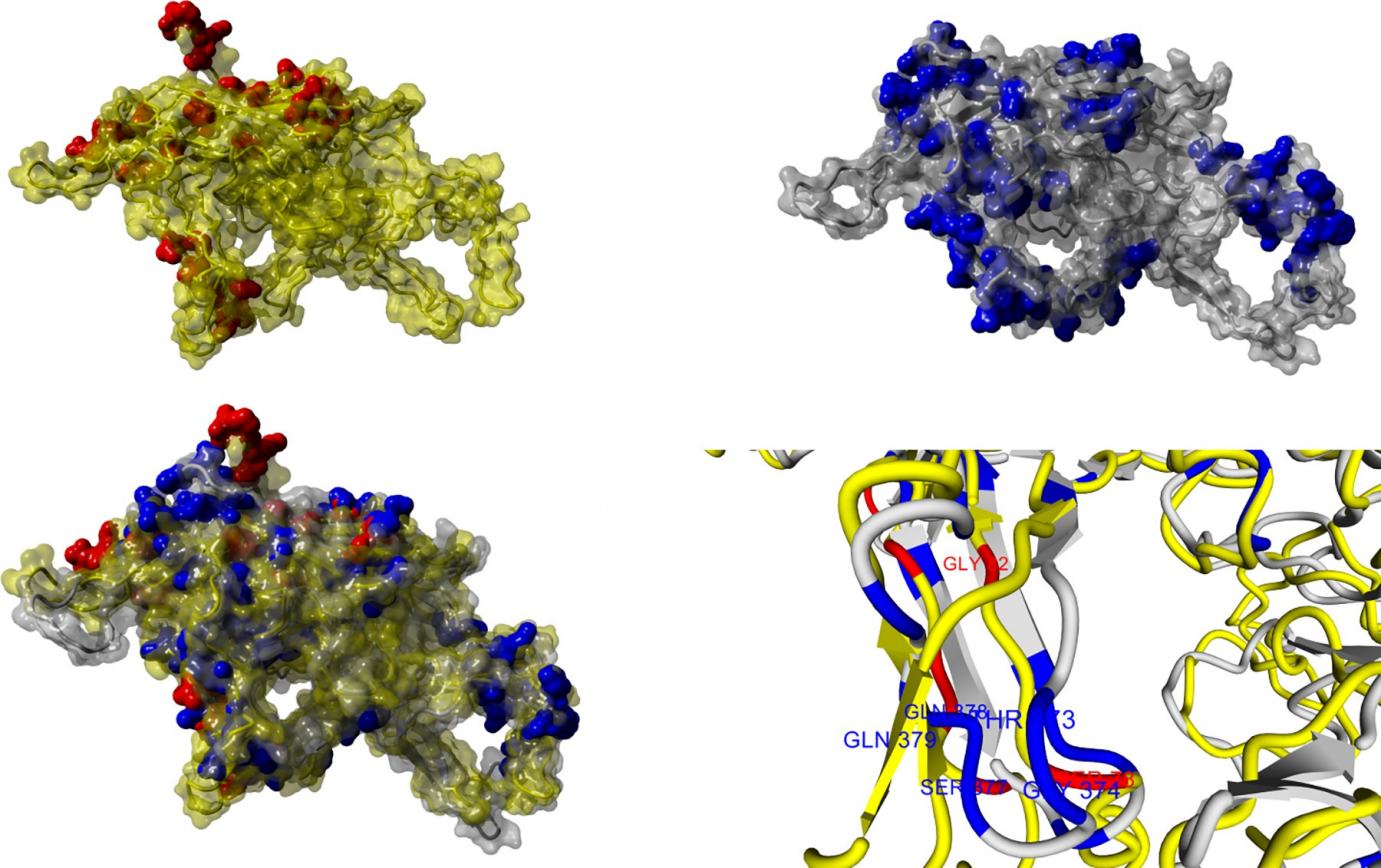
**a)** The 3D structure of the HPV-B19 capsid protein with red regions indicating the alignment of amino acids with a score greater than 8 **b)** The 3D structure of the AAV-4 capsid protein with blue regions indicating the alignment of amino acids with a score greater than 8 **c)** The alignment of both viral capsid proteins, with overlapping blue and red regions indicating amino acid alignment scores greater than 8. **d)** The overlapping regions of autoimmune encephalitis-associated amino acids in the HPV-B19 and AAV-4 viral capsid protein structures (GLY82, SER78, THR373, GLY374, SER377, GLN378, GLN379).

We used the MUSTANG algorithm to align the tertiary structure of the HPV-B19 and AAV-4 capsid proteins and MBP (Fig 7A and 7B). Aligned amino acids with a high score of 8 in Figs 5 and 6 were chosen for evaluation of their 3D position in the two viral capsid protein structures (Fig 8A–8C). The similarity in the aligned positions between the two virus structures is very noticeable on the left side of the images of the two 3D structures. According to the UniProt database, regions 179 to 222 and 246 to 256 have been identified as inducing experimental autoimmune encephalomyelitis (EAE) 1 and 2, respectively. When aligning the MBP sequence with the viral capsid protein structures, certain amino acids in these EAE-associated regions were found to have a score greater than 8. Interestingly, further examination of the 3D structures revealed that despite differences in amino acid sequence, the aligned regions were situated in the same area of the capsid protein structures. As illustrated in Fig 8D, the amino acids corresponding to EAE 1 in the 3D structure of the HPV-B19 viral capsid protein and those corresponding to EAE 2 in the 3D structure of the AAV-4 viral capsid protein were both located in the same region of the 3D structures. These findings suggest a possible conserved, structure-dependent mechanism of molecular mimicry and immune dysregulation in the development of autoimmune encephalitis.

### Similarity of sequences across microorganisms

#### Human herpes virus and MBP

In a separate example of potential molecular mimicry, we noted that both the *MDRPRTPPPSYSE* sequence of the U24 protein from HHV-6 and the similar *IVTPRTPPPSQGK* sequence of MBP can trigger a response by CD4+ T cells in MS patients [45].

We used this similarity in peptide sequence to search for additional pathogens that could potentially mimic the same epitope. The *PRTPPPS* sequence present in both proteins was subjected to a BLAST analysis, which yielded sequences from three fungi and one amoeba species (Table 4). To assess the immunogenicity of these sequences and their effect on CD4+ T cells in the immune system, the obtained results were analyzed in the Immune Epitope Database (IEDB), which combines prediction methods for major histocompatibility complex (MHC) binding (using the 7-allele method) [46], and immunogenicity. Since sequences in this database must be at least 15 amino acids long, we used sequences that extended beyond those shown in Table 4. We also examined scores obtained for human leucocyte antigen-DRB1:15:01 (HLA- DRB1:15:01), based on the previously established association between this allele and MS [47]. Table 5 displays the results of this analysis. The score for the MS-associated HLA-DRB:15:01 sequence is high in this analysis, supporting the biological relevance of the IEDB prediction (Table 5). Among the analyzed sequences, *Dictyostelium discoideum* has a higher immunogenicity score compared to the other sequences.

**Table 4:** BLAST Peptide of MBP sequence 230 to 236.

***Protein sequence (as a study input) and its position***						
Human	230	*PRTPPPS*			236	MBP
***Protein sequences found using BLAST and their positions***						
*Aspergillus clavatus* NRRL1	1124		*PSTPPPS*	1130		Protein transport protein sec31
*Aspergillus fischeri* NRRL 181	1110		*PSTPPPS*	1116		Protein transport protein sec31
*Aspergillus fumigatus* Af293	1110		*PSTPPPS*	1116		Protein transport protein sec31
*Dictyostelium discoideum*	95		*PPTPPPS*	101		Homeobox protein 12

**Table 5:** The prediction scores for CD4+ T cell immunogenicity of the analyzed sequences using the CD4+ T cell prediction tool in the IEDB. The scores for HLA-DRB1:15:01 allele is bolded and underlined as they are important in the association with MS.

*Protein Name*	*Organism*, *Amino acids position*	*Peptide*	*Combined Score*	*Immuno-genicity Score*	*Peptide core*	*Median Percentile Rank (7-allele)*	*HLA-DRB 1*:*03*:*01*	*HLA-DRB 1*:*07*:*01*	*HLA-DRB 1*:*15*:*01*	*HLA-DRB 3*:*01*:*01*	*HLA-DRB 3*:*02*:*02*	*HLA-DRB 4*:*01*:*01*	*HLA-DRB 5*:*01*:*01*
Protein transport protein sec31	*Aspergillus clavatus* 1122–1136	*YAPSTPPPSQLPMQQ*	87.84488	99.6122	*YAPSTPPPS*	80	93	73	**95**	77	73	82	80
	*Aspergillus fischeri*, *Aspergilllus fumigatus* 1108–1122	*YAPSTPPPSQLPMQQ*	87.84488	99.6122	*YAPSTPPPS*	80	93	73	**95**	77	73	82	80
Homeobox protein 12	*Dictyostelium discoideum* (Slime mold) 92–106	*QATPPTPPPSSSSLL*	99.99964	99.9991	*PTPPPSSSS*	100	100	63	**100**	94	100	94	100
U24 protein	human herpesvirus 6A	*MDRPRTPPPSYSEVL*	96.95668	99.8917	*DRPRTPPPS*	95	97	91	**92**	88	98	97	95
	1–15												
MBP	Human	*IVTPRTPPPSQGKGR*	97.43512	99.5878	*VTPRTPPPS*	96	88	98	**96**	97	95	97	83
	227–241												

### Mice gut flora and the MOG

We then utilized the reported similarities between MOG and *Lactobacillus and OUT002* protein sequences (Table 2) to perform a search for additional microorganisms encoding a similar peptide using the BLAST database. The results of this analysis are presented in Table 6. The findings indicate the presence of proteins highly similar to MOG in various bacteria. Several sequences were found from *Lactobacillus*, which is part of the gut flora and also plays a protective role in the sinus cavities, preventing sinusitis [48–50]. Additional sequences include the *Clostridium* elongating factor, G; a ribosomal protein with cation-dependent endonuclease activity from the bacterium *Leptotrix*, a bacterium abundant in running water containing iron and manganese [51]; a protein from *Oenococcus oeni*, a lactic acid bacterium abundant in butter [52]; 6-methylsalicylic acid synthase from *Aspergillus* species, which is involved in synthesis of patulin toxin [53, 54]; a protein from the thermophilic archaebacterium *Methanococcus* that performs methanogenic metabolism and participates in biochemical cycles [55]; and elongation factor G from *Penicillium* species. Two of the *Penicillium* species in Table 6 can also produce the hazardous patulin toxin, and one of these (known as blue mold), has been linked to apple contamination [56, 57], supporting pathogenicity for at least some of this group of proteins.

**Table 6:** BLAST results of bacterial proteins.

*Organism*	*Sequence information*			*Protein name*
*Aspergillus clavatus* (strain ATCC 1007 / CBS 513.65 / DSM 816 / NCTC 3887 / NRRL 1)	1468	*T* ***R*** *AG* ***F*** *T* ***RV*** *L*	1475	6-methylsalicylic acid synthase
*Methanocaldococcus jannaschii* (strain ATCC 43067 / DSM 2661 / JAL-1 / JCM 10045 / NBRC 100440) (*Methanococcus jannaschii*)	375	*K* ***R*** *DG* ***F*** *I* ***RV*** *L*	383	Adenine deaminase
*Lactobacillus sakei* subsp. *sakei* (strain 23K)	50	*L* ***R*** *DG* ***F*** *I* ***RV*** *R*	58	SsrA-binding protein
*Penicillium expansum (Blue mold rot fungus)*	1523	*T* ***R*** *DA* ***F*** *N* ***RV*** *L*	1531	6-methylsalicylic acid synthase
*Penicillium patulum (Penicillium griseofulvum)*	1523	*T* ***R*** *DA* ***F*** *N* ***RV*** *L*	1531	6-methylsalicylic acid synthase
*Lactobacillus plantarum*	172	*K* ***R*** *EG* ***F*** *V* ***RV*** *R*	180	UvrABC system protein A (UVRA_LACPL)
*Oenococcus oeni* (strain ATCC BAA-331 / PSU-1)	332	*G* ***R*** *LT* ***F*** *L* ***RV*** *Y*	340	Elongation factor G
*Lactobacillus fermentum* (strain NBRC 3956 / LMG 18251)	324	*G* ***R*** *LT* ***F*** *L* ***RV*** *Y*	332	Elongation factor G
*Clostridium perfringens* (strain ATCC 13124 / DSM 756 / JCM 1290 / NCIMB 6125 / NCTC 8237 / Type A)	321	*G* ***R*** *LA* ***F*** *T* ***RV*** *Y*	329	Elongation factor G
*Leptothrix cholodnii* (strain ATCC 51168 / LMG 8142 / SP-6) (*Leptothrix discophora* (strain SP-6))	50	*L* ***R*** *IA* ***F*** *D* ***RV*** *E*	58	30S ribosomal protein S16

### MAP, EBV, IRF5 proteins and MBP

**Fig 9:**
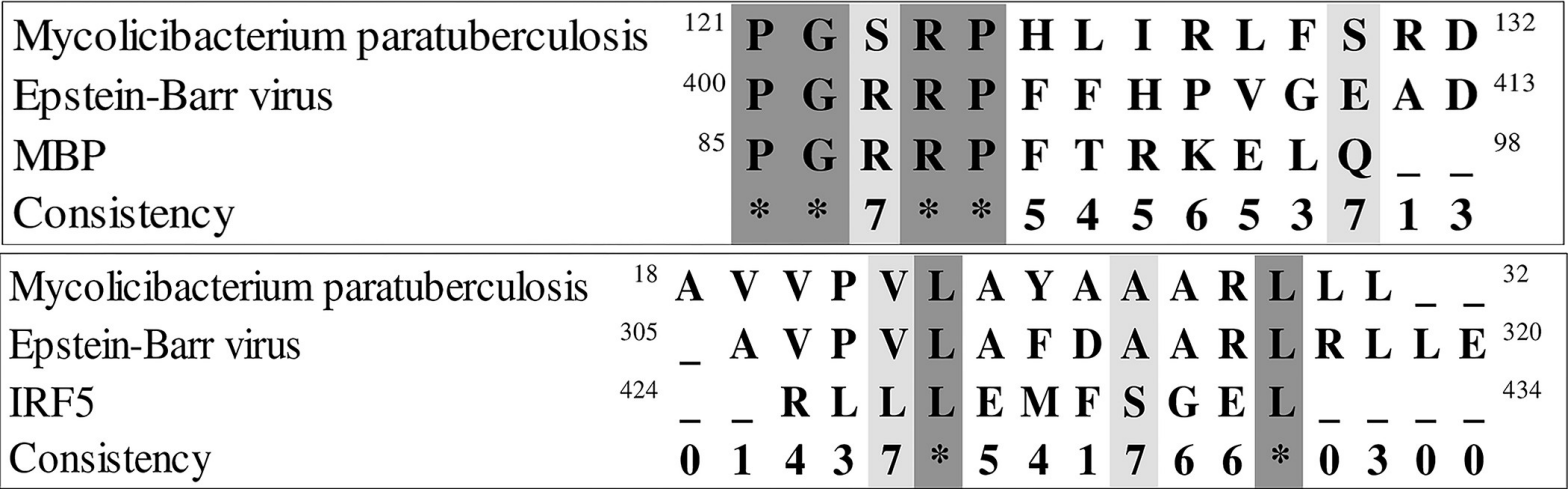
**a)** Multiple alignments of MAP_0106, EBNA1, and MBP proteins. **b)** Multiple alignments of MAP_4027، BOLF1, and IRF5 proteins.

**Fig 10:**
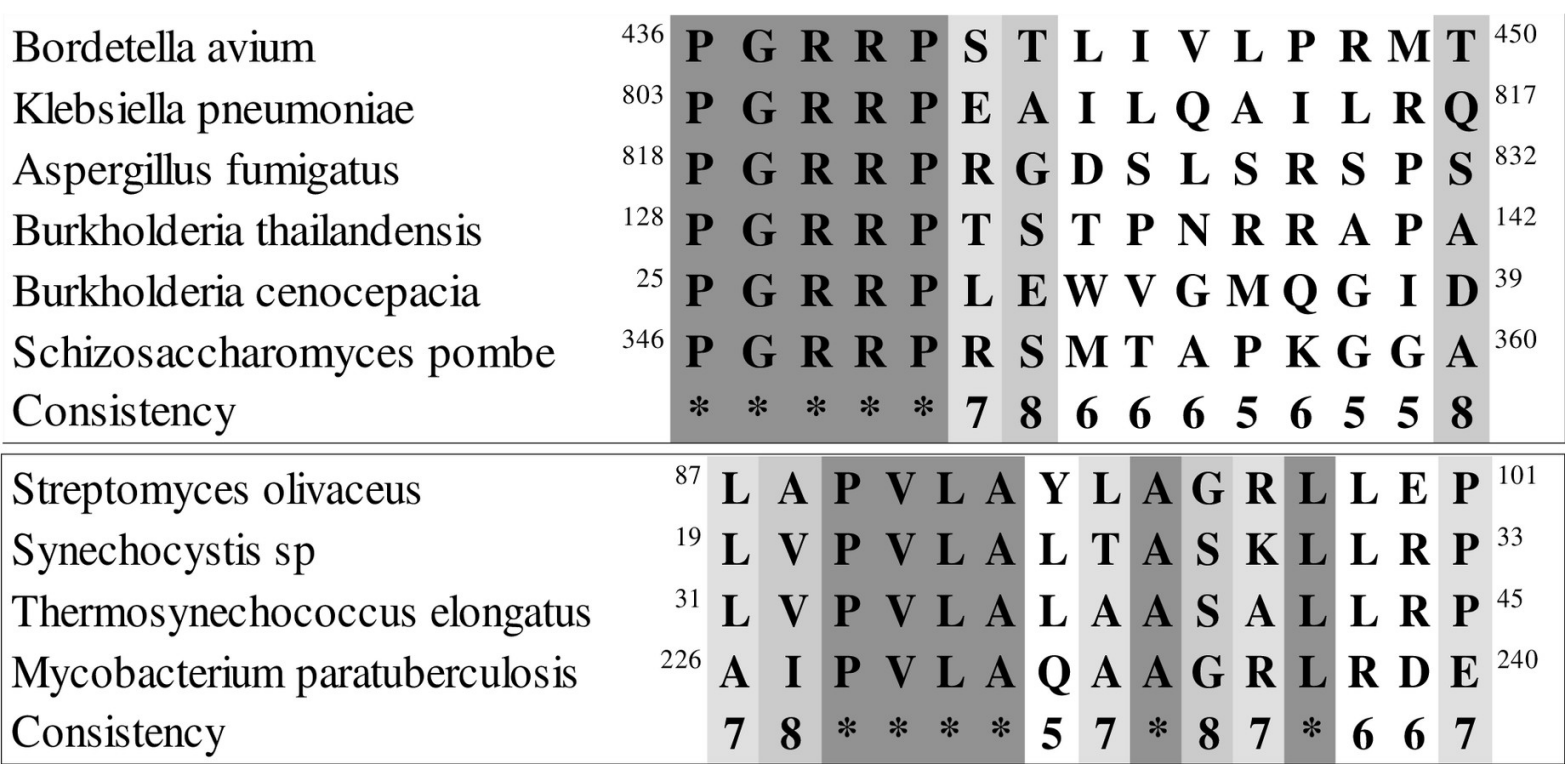
The multiple alignment results based on the two categories presented in Table 7. **a)** shows the multiple alignment results of the first group of BLAST protein sequences (EBNA1, MAP 0106, MBP) **b)** shows the multiple alignment results of the second group of BLAST protein sequences (BOLF1, IRF5, MAP4027).

We performed an analysis using the BLAST database to identify additional hits for the peptide sequences with alignments between *Mycolicibacterium paratuberculosis*, Epstein-Barr virus, and *Homo sapiens* (Table 1). The resulting hits were aligned with each of the two sets of similar sequences (Fig 9). We analyzed the protein sequences and conserved sites of each protein (similarity set 1. MAP 0106, EBNA1, and MBP; similarity set 2. MAP 4027, BOLF1, and IRF5) using BLAST (Fig 10 and Table 7). The results reveal additional pathogens with sequences similar to the two groups of MAP and EBV peptide sequences. These pathogens include a species of *Aspergillus* fungus that causes lung infection [58], where similarity was found for a protein responsible for editing pre-mRNA was identified, and *Klebsiella pneumonia*, a bacterium that forms part of the normal human mouth, skin, and intestines flora and can cause lung alveoli infections and pneumonia in people with weakened immune systems, where similarity was found to a protein-synthesizing pyrroloquinoline quinone [59]. Similarity to a bromodomain-containing protein from *Schizosaccharomyces pombe*, the common model organism, was also found [60].

**Table 7:** The BLAST results for two categories of protein sequences: 1. MAP 0106, EBNA1, and MBP and 2. MAP 4027, BOLF1, and IRF5.

***Results obtained from the first group (EBNA1*, *MAP_0106*, *MBP)***					***HLA-DRB 1*:*15*:*01 Score***
***Organism***	***Sequence information***			***Protein name***	
*Aspergillus fumigatus* (strain ATCC MYA-4609 / Af293 / CBS 101355 / FGSC A1100)	818	*PGRRPRGDSLSRSPS*	832	Pre-mRNA-splicing factor cwc22	97
*Klebsiella pneumoniae*	503	*PGRRPEAILQAILRQ*	517	Coenzyme PQQ synthesis protein F	35
*Schizosaccharomyces pombe* (strain 972 / ATCC 24843) (Fission yeast)	346	*PGRRPRSMTAPKGGA*	360	Bromodomain-containing protein C631.02	82
*Burkholderia thailandensis* (strain ATCC 700388 / DSM 13276 / CIP 106301 / E264)	128	*PGRRPTSTPNRRAPA*	142	Endoribonuclease YbeY	93
*Burkholderia cenocepacia* (strain MC0-3)	25	*PGRRPLEWVGMQGID*	39	GTP cyclohydrolase FolE2 2	60
*Bordetella avium* (strain 197N)	436	*PGRRPSTLIVLPRMT*	450	Glucose-6-phosphate isomerase	43
**Results obtained from the second group (BOLF1, IRF5, MAP4027)**					
**Organism**	**Sequence information**			**Protein name**	
*Mycobacterium paratuberculosis* (strain ATCC BAA-968 / K-10)	227	*AIPVLAQAAGRLRDE*	241	Trehalose-phosphate phosphatase	13
*Synechocystis* sp. (strain PCC 6803 / Kazusa)	19	*LVPVLALTASKLLRP*	33	NAD(P)H-quinone oxidoreductase subunit 3	3.6
*Streptomyces olivaceus*	87	*LAPVLAYLAGRLLEP*	101	Elloramycin glycosyltransferase ElmGT	0.41
*Thermosynechococcus vestitus* (strain NIES-2133 / IAM M-273 / BP-1)	31	*LVPVLALAASALLRP*	45	NAD(P)H-quinone oxidoreductase subunit 3	4.3

Two proteins from *Borkhdelriaia* species were also found to have similarity to the MBP antigen. *B*. *thailandensis* is used to model infection of other species of this bacterium family since it is easier to work with and does not require high-level biological facilities. *B*. *cenocepacia* causes lung infections in cystic fibrosis patients and is one of the most dangerous pathogens for these patients [61, 62]. *Bordetella avium*, the final bacterium in the first group, causes a respiratory infection in turkeys by attaching to and spreading to respiratory epithelial cells, a disease is known as Bordetellosis, and is also considered an opportunistic pathogen in patients with cystic fibrosis [63, 64]. A portion of the enzyme glucose 6-phosphate isomerase from this bacterium was found to have similarity to the MBP epitope.

**Fig 11:**
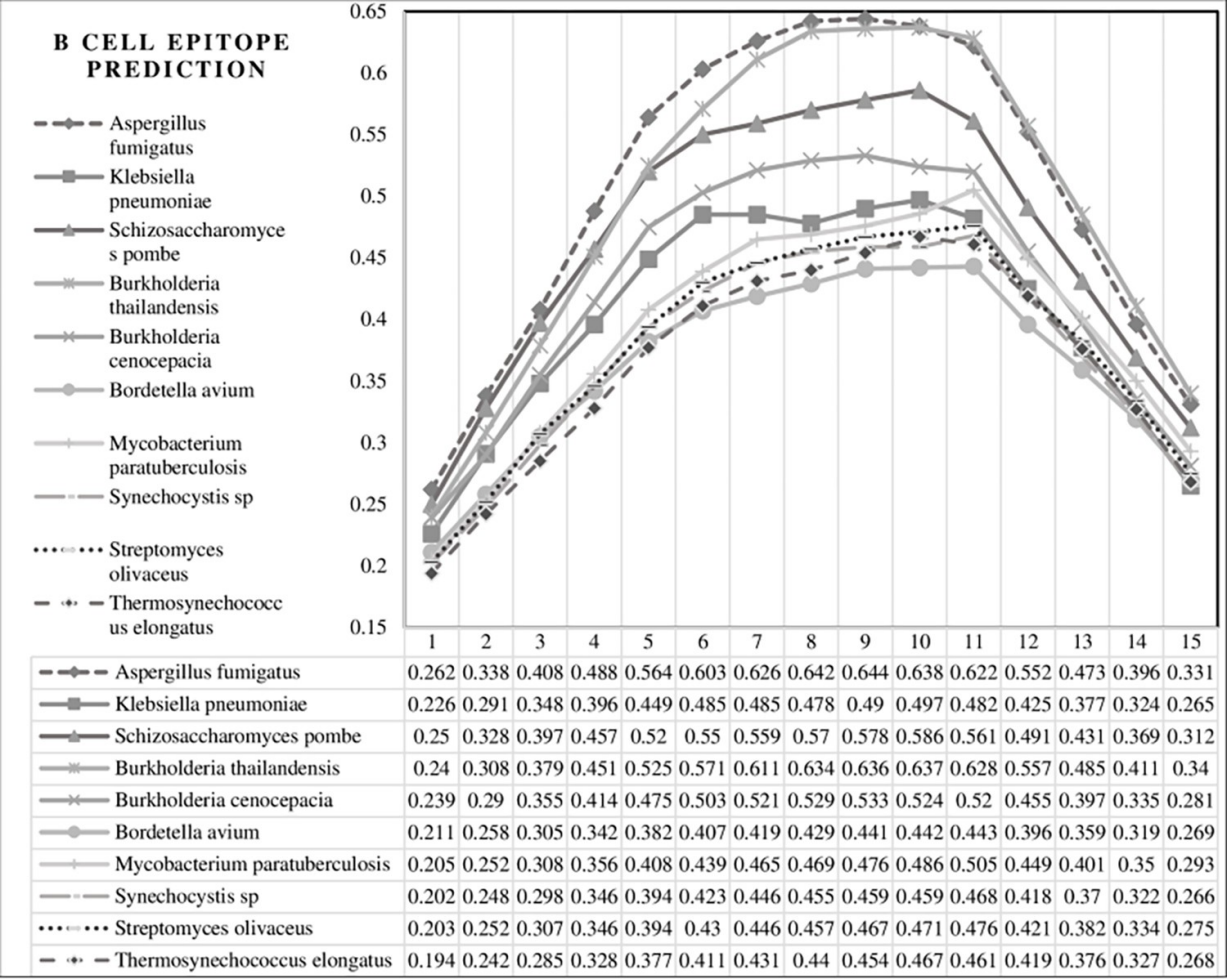
Diagram of the sequences of epitope antigens obtained from Table 7.

In the second alignment cluster, Trehalose Phosphate Phosphatase from the bacterium MAP, causes tuberculosis and respiratory infections, was identified [65]. The analysis also identified an NAD(P)H-quinone oxidoreductase enzyme from two cyanobacteria, the freshwater single-celled cyanobacterium *Synechocystis* sp. PCC6803 [66] and *Thermosynechococcus elongatus*. The elmGT protein of *Streptomyces* bacteria, which is involved in the biosynthesis of alaromycin and glucose transport, was also identified. Table 7 also shows the probability percentage of each sequence as an epitope of an antigen in CD4+ T cell activation specifically in MS, based on the leukocyte antigen allele HLA-DRB1:15:01 and determined by the IEDB [67]. A previous study also analyzed these sequences for their potential to elicit an immune response in B cell antibodies (Fig 11 and Table 8). The amino acid scores for each sequence are presented in Fig 11, where the horizontal axis of this diagram represents the position of amino acids within each sequence, while the vertical axis represents the corresponding scores for each amino acid.

**Table 8:** The sequences of the antigen as an epitope structure. Each amino acid from the sequences has a corresponding score in the epitope structure, which is shown in **Fig 11**. Amino acids with scores higher than the threshold (0.5) in this prediction tool are bolded.

*Aspergillus fumigatus*	*Klebsiella pneumoniae*	*Schizosaccharomyces pombe*	*Burkholderia thailandensis*	*Burkholderia cenocepacia*	*Bordetella avium*	*Mycobacterium paratuberculosis*	*Synechocystis* sp	*Streptomyces olivaceus*	*Thermosynechococcus elongatus*
*P*	*P*	*P*	*P*	*P*	*P*	*A*	*L*	*L*	*L*
*G*	*G*	*G*	*G*	*G*	*G*	*I*	*V*	*A*	*V*
*R*	*R*	*R*	*R*	*R*	*R*	*P*	*P*	*P*	*P*
*R*	*R*	*R*	*R*	*R*	*R*	*V*	*V*	*V*	*V*
***P***	*P*	***P***	***P***	*P*	*P*	*L*	*L*	*L*	*L*
***R***	*E*	***R***	***T***	***L***	*S*	*A*	*A*	*A*	*A*
***G***	*A*	***S***	***S***	***E***	*T*	*Q*	*L*	*Y*	*L*
***D***	*I*	***M***	***T***	***W***	*L*	*A*	*T*	*L*	*A*
***S***	*L*	***T***	***P***	***V***	*I*	*A*	*A*	*A*	*A*
***L***	*Q*	***A***	***N***	***G***	*V*	*G*	*S*	*G*	*S*
***S***	*A*	***P***	***R***	***M***	*L*	***R***	*K*	*R*	*A*
***R***	*I*	*K*	***R***	*Q*	*P*	*L*	*L*	*L*	*L*
*S*	*L*	*G*	*A*	*G*	*R*	*R*	*L*	*L*	*L*
*P*	*R*	*G*	*P*	*I*	*M*	*D*	*R*	*E*	*R*
*S*	*Q*	*A*	*A*	*D*	*T*	*E*	*P*	*P*	*P*

### Correlation of epsilon toxin with MS

**Fig 12:**
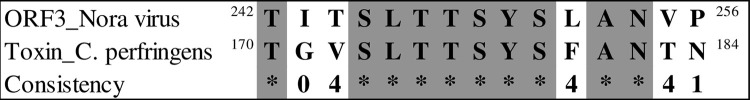
Alignment of epsilon toxin of *Clostridium perfringens* and ORF3 protein of Nora virus.

**Fig 13:**
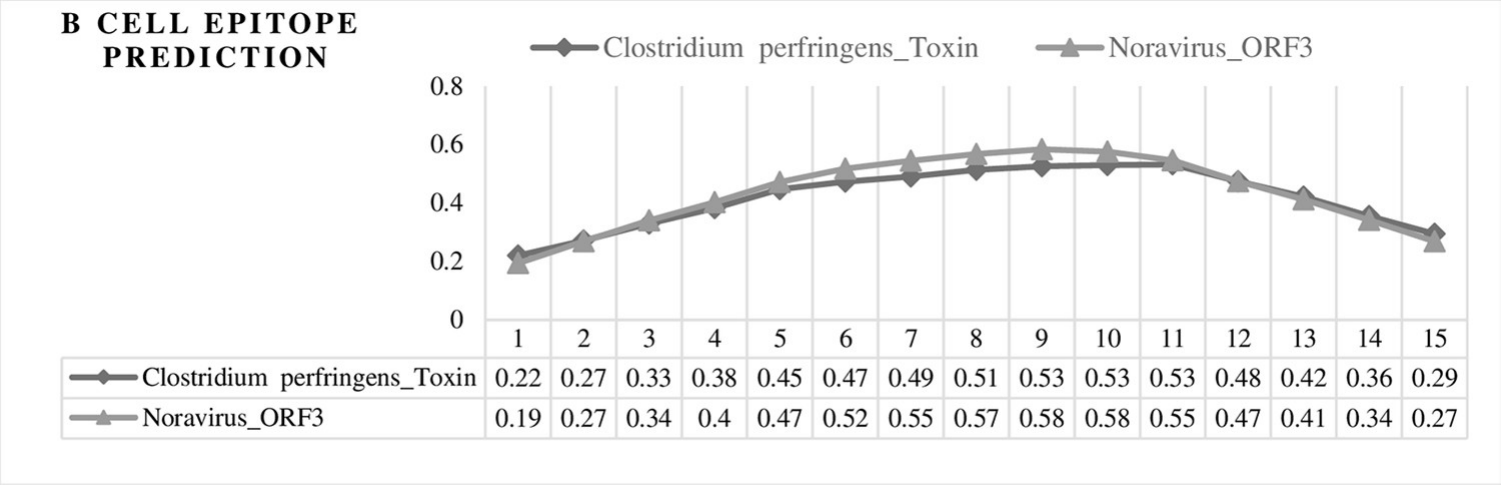
Sequence analysis of epsilon toxin and ORF3 proteins as an antigen epitope. The diagram in this figure shows the position of amino acids in each sequence on the horizontal axis and the score of each amino acid on the vertical axis. Also, the amino acids are shown separately, in which their score of them are higher than the threshold, and they were bolded (Table 9).

Epsilon toxin from *Clostridium perfringens* has been previously linked to MS primarily based on its known neurotoxic effects and the presence of antibodies against epsilon toxin in some MS patients. The amino acid sequence *TGVSLTTSYSFANTN* (170–184) of the epsilon protein has been specifically implicated in MS. Using BLAST, a similar sequence was obtained from Nora virus. The two sequences aligned well with each other (Fig 12). The UniProt database indicates that this similar sequence is hosted by the *Drosophila* Uncharacterized ORF3 protein, which is a member of the Picornavirus family that can infect the intestines of all *Drosophila* species. The epitope score from IEDB for the antigenic regions of the Nora virus was found to be higher than bacterial epsilon sequences. The amino acids were individually investigated, and their scores were part of the antigen epitope (Fig 13).

**Table 9:** Antigen epitopes derived from Nora virus and *Clostridium perfringens*-related sequences.

*Position*	*Clostridium perfringens*	*Nora virus*
1	*T*	*T*
2	*G*	*I*
3	*V*	*T*
4	*S*	*S*
5	*L*	*L*
6	*T*	***T***
7	*T*	***T***
8	***S***	***S***
9	***Y***	***Y***
10	***S***	***S***
11	***F***	***L***
12	*A*	*A*
13	*N*	*N*
14	*T*	*V*
15	*N*	*P*

These findings suggest that the toxin might play a role in MS pathology, although the evidence is not conclusive. Alternatively, this could represent a case of incidental cross-reactivity, where the immune system reacts to epsilon toxin without it being a causative factor in MS.

### Spike and protease of SARS-CoV-2

**Fig 14:**
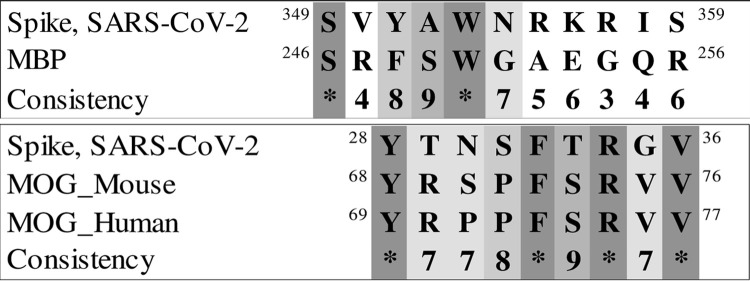
a) Sequence alignment of MBP 246–256 with spike glycoprotein 349–359. b) Sequence alignment of 68 to 76 MOG with 28 to 36 spike glycoproteins.

**Fig 15:**
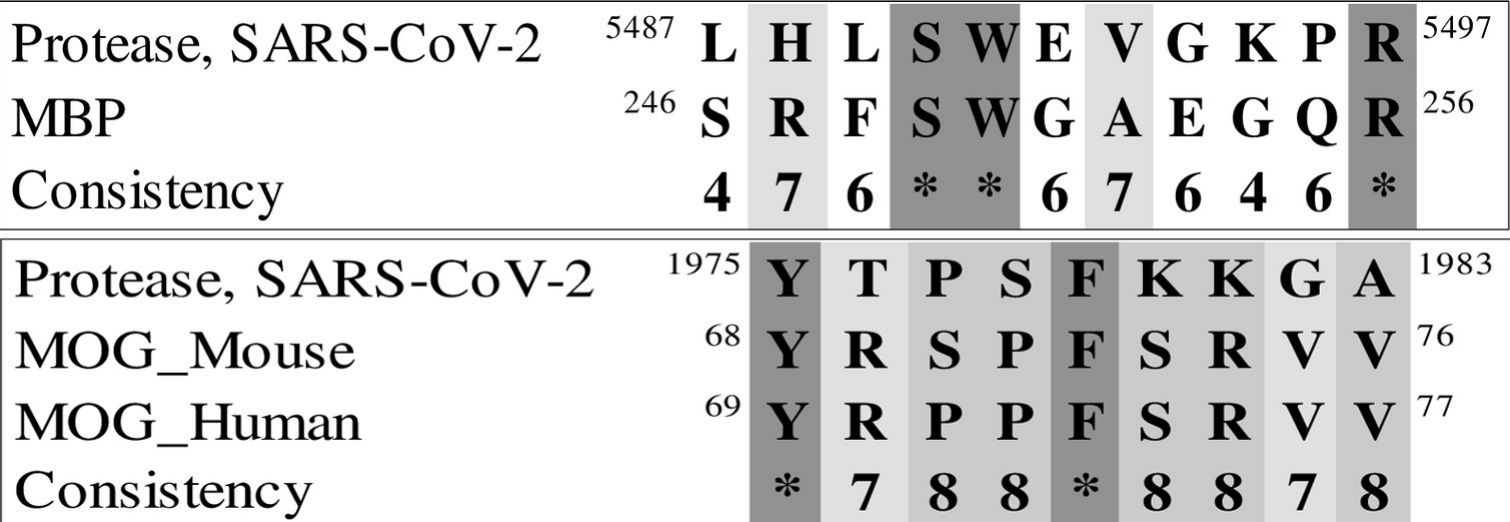
a) Sequence alignment of MBP 246–256 with main protease 5487–5497. b) Sequence alignment of 68 to 76 MOG with 1975 to 1983 main protease.

We next explored the similarities between two SARS-CoV-2 proteins and parts of MOG and MBP. First, the sequence similarity of MOG and MBP with the spike glycoprotein of the virus (UniProt ID: P0DTC2) was investigated (Fig 14). Next, alignment of MOG and MBP sequences with the SARS-CoV-2 main protease sequence (UniProt ID: P0DTD1) was carried out (Fig 15).

### Gut microbiota balance

Molecular mimicry between microbial and myelin proteins could initiate or exacerbate autoimmune responses in MS. Therefore, we next examined potential similarities between specific segments of the MOG and MBP sequences and the bile salt hydrolase (BSH) enzyme, and as well as several proteins involved in the metabolism of polysaccharide A was investigated.

### BSH enzyme and butyric acid metabolism

**Fig 16:**
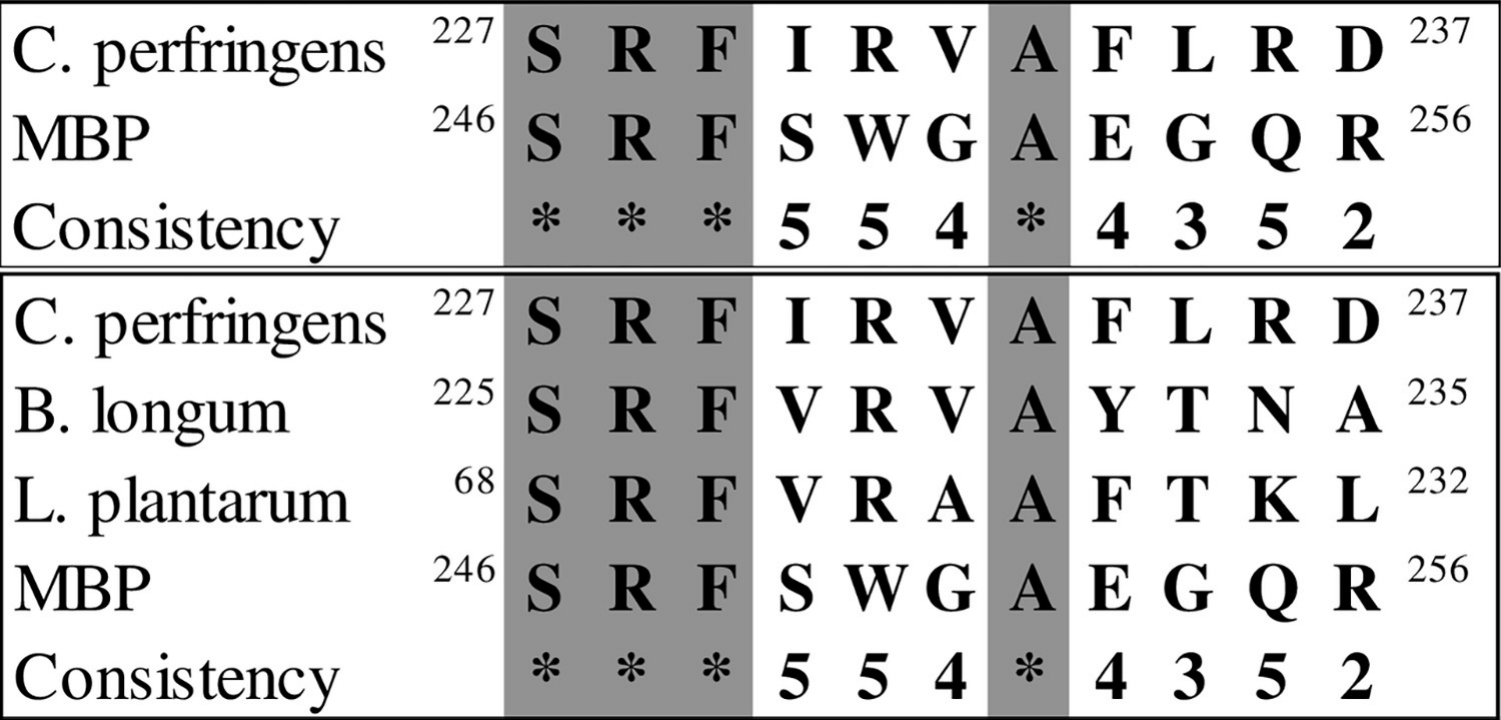
**a)** Alignment of *C*. *perfringens* BSH enzyme sequences (227–237) and MBP (from 246 to 256). **b)** Protected points in the alignment of three bacterial sequences of *C*. *perfringens*, *B*. *longum*, *L*. *plantarum*, and MBP.

We aligned positions 246 to 256 of MBP (which can cause autoimmune encephalitis) and 68 to 76 of MOG (four protected amino acids in a study of mice gut flora by Miyauchi et al. [29]) with the BSH enzyme from various bacterial species present in the gut flora. Specifically, using the data from Fig 3 we aligned the MBP sequence (246 to 256) with the C. perfringens BSH enzyme sequence (Fig 16A) and conducted multiple alignments of MBP sequences with bacterial sequences, including B. longum and L. plantarum (Fig 16B). The results of aligning the active site of the enzyme in a protected part of the sequence are shown in Fig 16. The results revealed the noteworthy presence of *Lactobacillus* in the alignment.

**Fig 17:**
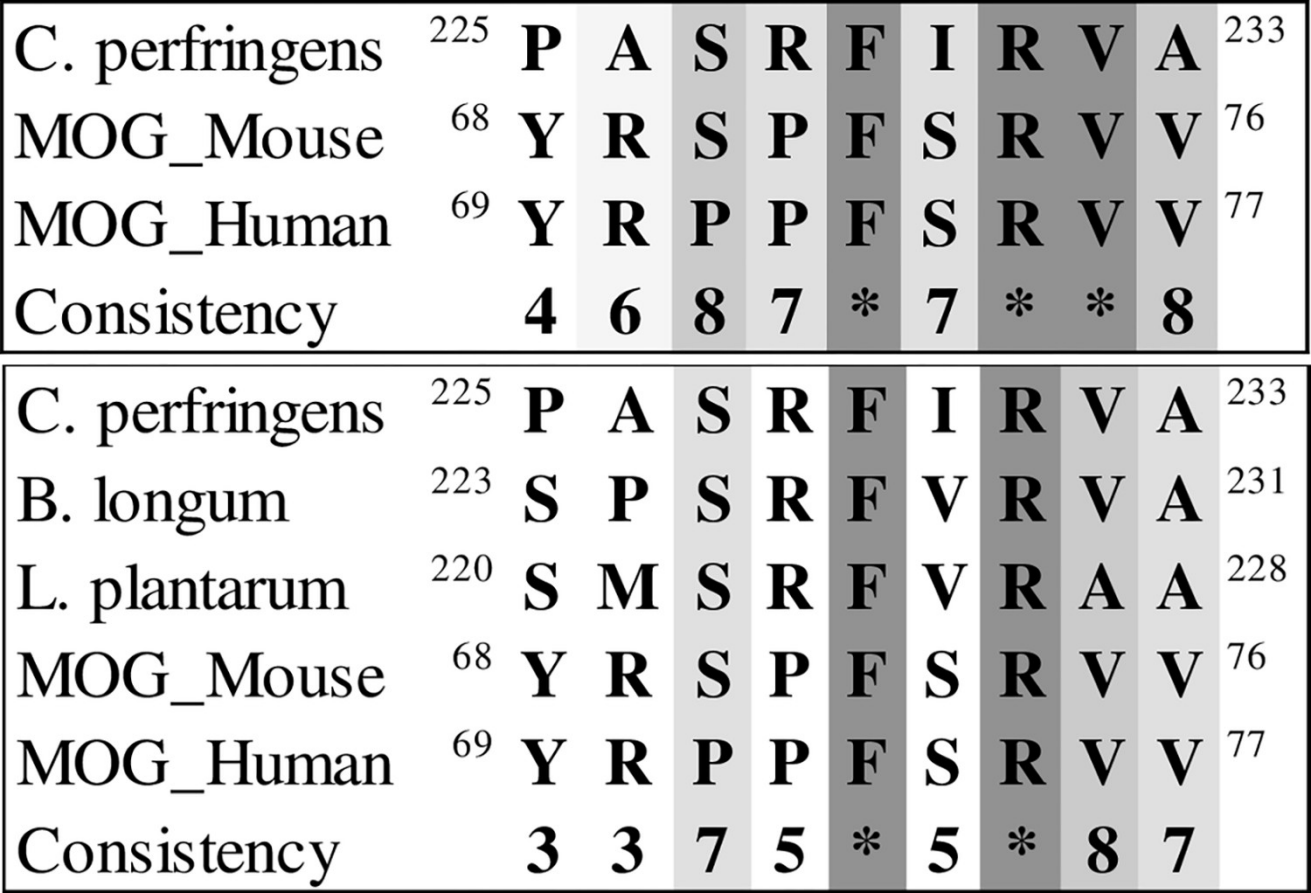
**a)** Alignment of human and mouse MOG with BSH protein from *C*. *perfringens*. **b)** Multiple alignments of MOG with the BSH proteins from *C*. *perfringens*, *B*. *longum*, *and L*. *plantarum*.

**Fig 18:**
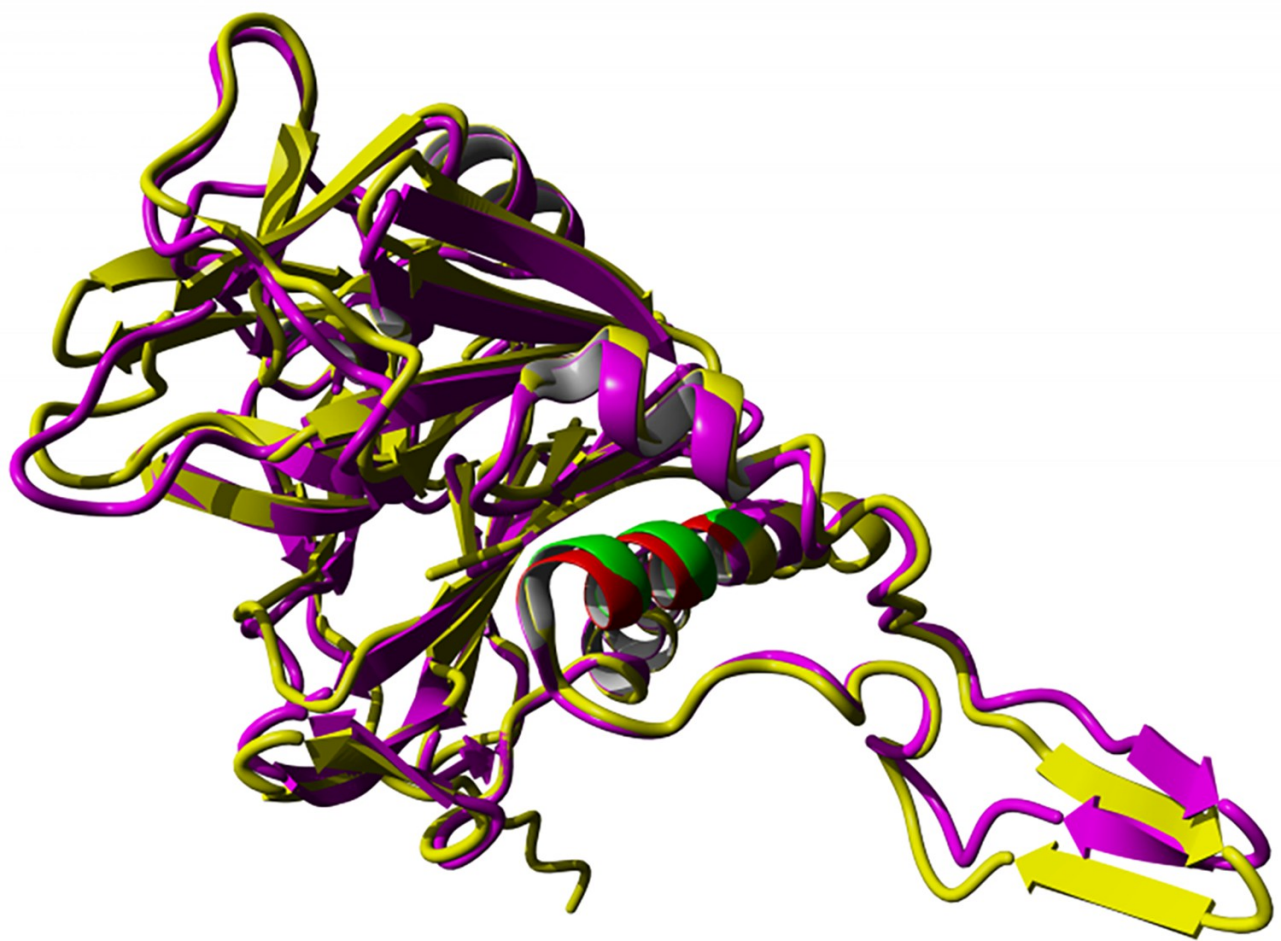
3D structure alignment and motif analysis of *B*. *Longum* (purple and red) and *C*. *Perfringens* (yellow and green) BSH proteins with MOG sequence.

In addition to the targeted alignment with MBP, the entire sequence of this protein from the bacterium was aligned with the MOG of mouse and human (Fig 17A). Notably, three out of four protected amino acids in the obtained results were found to be identical (according to list of Table 1). After determining the precise location of the MOG and *C*. *perfringens* alignment, a subsequent alignment with the other bacteria (*B*. *longum*, *L*. *plantarum*) was performed (Fig 17B). The 3D structure of two proteins of *B*. *longum* (PDB ID: 2HF0) and *C*. *perfringens* (PDB ID: 2RF8), determined by the X-ray diffraction method, exhibited a high degree of similarity (Fig 18). The 3D structure alignment of *B*. *longum and C*. *perfringens* are shown in purple and yellow, respectively. Fig 18 also highlights specific regions in red and green that correspond to motifs from the *B*. *longum* and *C*. *perfringens* proteins. These motifs were obtained from the result of the sequence alignment of these proteins with the MOG sequence. It is noteworthy that the sequence similarity between the two bacterial proteins is 37%. The alignment of the tertiary structure of these two proteins displayed a significant overlap, and the RMSD between two chains of molecules was 3.53, as calculated for Cα atoms, indicating high similarity.

### Polysaccharide A in *Bacteroides Fragilis*

**Fig 19:**
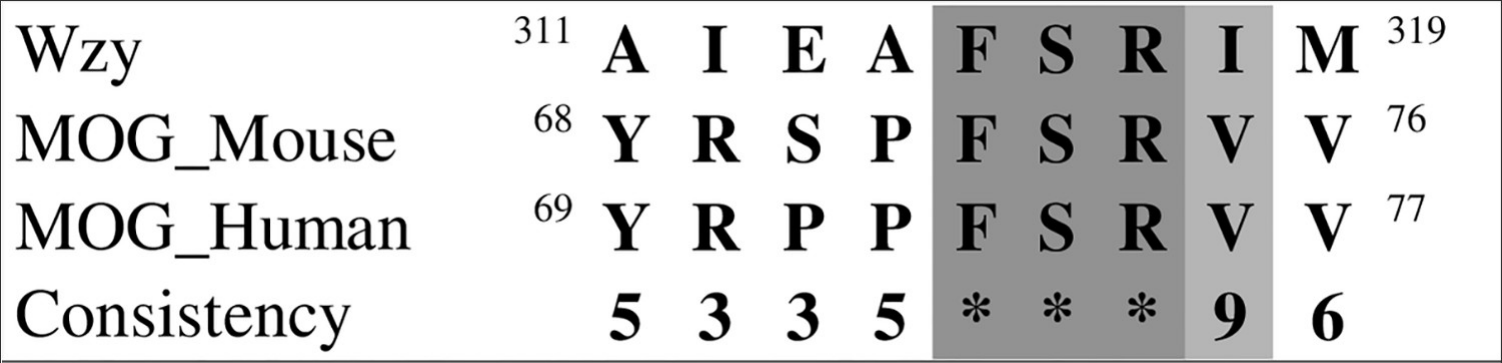
Relationship between MOG and the Wzy enzyme.

**Fig 20:**
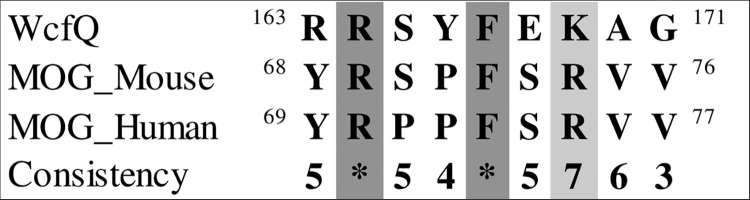
MOG (mouse and human) alignment with the bacterial enzyme WcfQ.

Finally, we found that certain segments of the MOG sequence bear resemblance to corresponding regions in two proteins implicated in polysaccharide A metabolism. Using the data from Fig 4 MOG was aligned with Wzy, an enzyme of bacterial origin that functions as a polymerase in the periplasmic compartment to facilitate polysaccharide subunit binding (Fig 19), and with glycosyltransferase (WcfQ) (Fig 20). Determination of tertiary structure similarity between these proteins has not been completed.

## Discussion

In this study, we analyzed public data to identify relationships between proteins or metabolites from microorganisms and the myelin proteins MBP and MOG, since such similarities may represent cases of molecular mimicry that contribute to autoimmunity and MS pathogenesis. We further considered the possibility that microorganism-encoded proteins with known links to MS may have structural similarity with proteins from other microorganisms and even other proteins from the same microorganism. A significant similarity in the secondary structure between HPV-B19 and AAV-4 with MBP was noted, despite sequence differences, highlighting the importance of conservation of secondary structure. Even minimal structural resemblances between MBP, MOG, and pathogenic agents may be sufficient to trigger cross-reactivity, suggesting shared functional motifs. In our comparison of HPV-B19 and AAV-4 capsid proteins with MBP (Fig 8C) we found that, in addition to sequence similarity, three different parts of the tertiary structure of the viral capsid proteins overlap with the MBP structure. Based on the presence of these motifs on the surface of capsid proteins, the observed sequence similarity in the alignment of these motifs, and the potential for the immune system to misidentify them, further laboratory investigations are necessary to understand the role of these antigens in development of MS.

The discovery of conserved molecular mimicry and immune dysregulation mechanisms between HPV-B19 and AAV-4 viruses in the development of autoimmune encephalitis (Fig 8D) raises the possibility that these viral structures may be involved in the initiation and perpetuation of the autoimmune response seen in some individuals with this disease. Understanding the molecular mechanisms underlying autoimmune encephalitis is crucial to developing effective therapies for this debilitating disease.

Antigens that are predicted to stimulate CD4+ cells of the immune system are significant for understanding MS. Three of the pathogens that encode antigens similar to MBP are *Aspergillus fischeri*, whose genome is very similar to *Aspergillus fumigatus*, which causes Aspergillosis [68]; *Aspergillus clavatus*, used in the production of enzymes [69]; and the soil amoebae *Dictyostelium discoideum*, which is used as a model in immune system studies [70]. All three of these organisms are pathogenic in humans, and also have applications for research and industrial activity. The high score of *Dictyostelium* compared to the HHV-6 sequence and MBP, predicting CD4+ cell activation and HLA-DRB:15:01 involvement, is noteworthy. All cases scored higher than the HHV-6 sequence itself.

In the case of the MOG sequence, two *Penicillium* species and one *Aspergillus* species were found to produce similar antigens, including a 6-methylsalicylic acid synthase, involved in patulin biosynthesis, and multiple instances of Elongation factor G. Proteins from *Aspergillus fumigatus*, *Schizosaccharomyces pombe*, and *Burkholderia thailandensis* scored high for predicting CD4+ activation based on the HLA-DRB1:15:01 allele. These proteins were also predicted in IEDB as antigen epitopes for B cells (Fig 11). Together, this information suggests that these three antigenic sequences have the potential to cause immune system cross-reactivity.

Extensive research on murine coronavirus was conducted prior to the COVID-19 pandemic, specifically on the effect of this coronavirus on the nervous system of mice and the induction of encephalomyelitis. In these studies, demyelination in neurons is structurally similar to human MS disease [71–73]. Murine and SARS-CoV-2 viruses belong to the same genus, and new research shows that SARS-CoV-2 remains in the human brain [74]. Based on our sequence alignments between part of MOG and MBP proteins with the spike and protease proteins of the virus, amino acid similarity is exceptionally high for MOG (Figs 14B and 15B). These motifs are located on the surface of the virus proteins in their 3D structure, and could contribute to triggering autoimmunity. Further investigation is warranted to understand the mechanism and effects of this similarity.

This study also identified similarities in the sequences of MBP and MOG with the gut flora enzymes BSH, WcfQ, and Wzy. The findings suggest that an imbalance in the human microbial flora can disrupt metabolite production, potentially leading to biological effects in the body such as in the CNS. Further research is needed to fully understand the link between the human intestinal microbiota and autoimmune conditions.

MBP has been extensively studied in the context of MS, and there is a vast body of literature available on its role in the disease [3, 75]. Our approach was focused on exploring the potential relationship between conserved secondary structures of proteins. We observed a high degree of similarity between the secondary structure of HPV-B19 and AAV-4 with MBP. While there is not always a high degree of primary sequence similarity between MBP and MOG and the proteins encoded by these pathogens, it is important to note that the secondary structure is conserved and is critical to the function of the protein. Therefore, even small similarities in structure between both MBP and MOG and antigenic proteins from pathogens could be significant in terms of understanding potential interactions. There may be motifs and specific regions in the structure of other pathogenic proteins that are similar to those in MBP and MOG. By checking similarities in these structures against reported information on the association between the specific pathogens and MS disease, we can potentially identify specific factors and uncover new relations between MOG and MBP in humans and microorganismal pathogenic agents.

## Conclusion

The immune system may mistakenly cross-identify pathogen-encoded and human proteins, contributing to autoimmunity. We show that molecular mimicry of the myelin proteins MBP and MOG by pathogen-encoded proteins can occur due to relatively short, separated amino acid sequences or 3D structure, not just amino acid sequence conservation. We identify additional species and proteins that may also represent cases of molecular mimicry and contribute to MS pathogenesis. We emphasize the importance of investigating the complex relationship between the human intestinal microbiota, viral infections like HPV-B19, AAV-4, and COVID-19, and the development of autoimmune disorders.

## Supporting information

S1 Appendix (PDF)

## Abbreviations

3D: three-dimensional
AAV-4: adeno-associated virus 4
BepiPred-2.0: Bepipred Linear Epitope Prediction 2.0
BLAST: Basic Local Alignment Search Tool
BSH: bile salt hydrolase
CD4: cluster of differentiation 4
CNS: central nervous system
EAE: experimental autoimmune encephalomyelitis
EBV: Epstein-Barr virus
EMBL: European Molecular Biology Laboratory
HHV-6: human herpesvirus 6
HERV: human endogenous retroviruses
HLA: human leukocyte antigens
HPV-B19: human parvovirus B19
IEDB: Immune Epitope Database
IRF-5: interferon regulatory factor 5
MAP: mycobacterium paratuberculosis
MBP: myelin basic protein
MOG: myelin oligodendrocytes glycoprotein
MS: multiple sclerosis
NCBI: National Center for Biotechnology Information
PDB: Protein Data Bank
RCSB: Research Collaboratory for Structural Bioinformatics
RMSD: root-mean-square deviation
WcfQ: glycosyltransferase
