# Supplementary material for: Bioinformatics analysis of myelin-microbe interactions suggests multiple types of molecular mimicry in the pathogenesis of multiple sclerosis
Source: PLoS One. 2024 Dec 30;19(12):e0308817. doi: 10.1371/journal.pone.0308817 (PMC11684644; doi:10.1371/journal.pone.0308817)
Supplement: S1 Appendix — (PDF) [file pone.0308817.s001.pdf]

Detailed insights into capsid alignments (Fig. 8 Results)

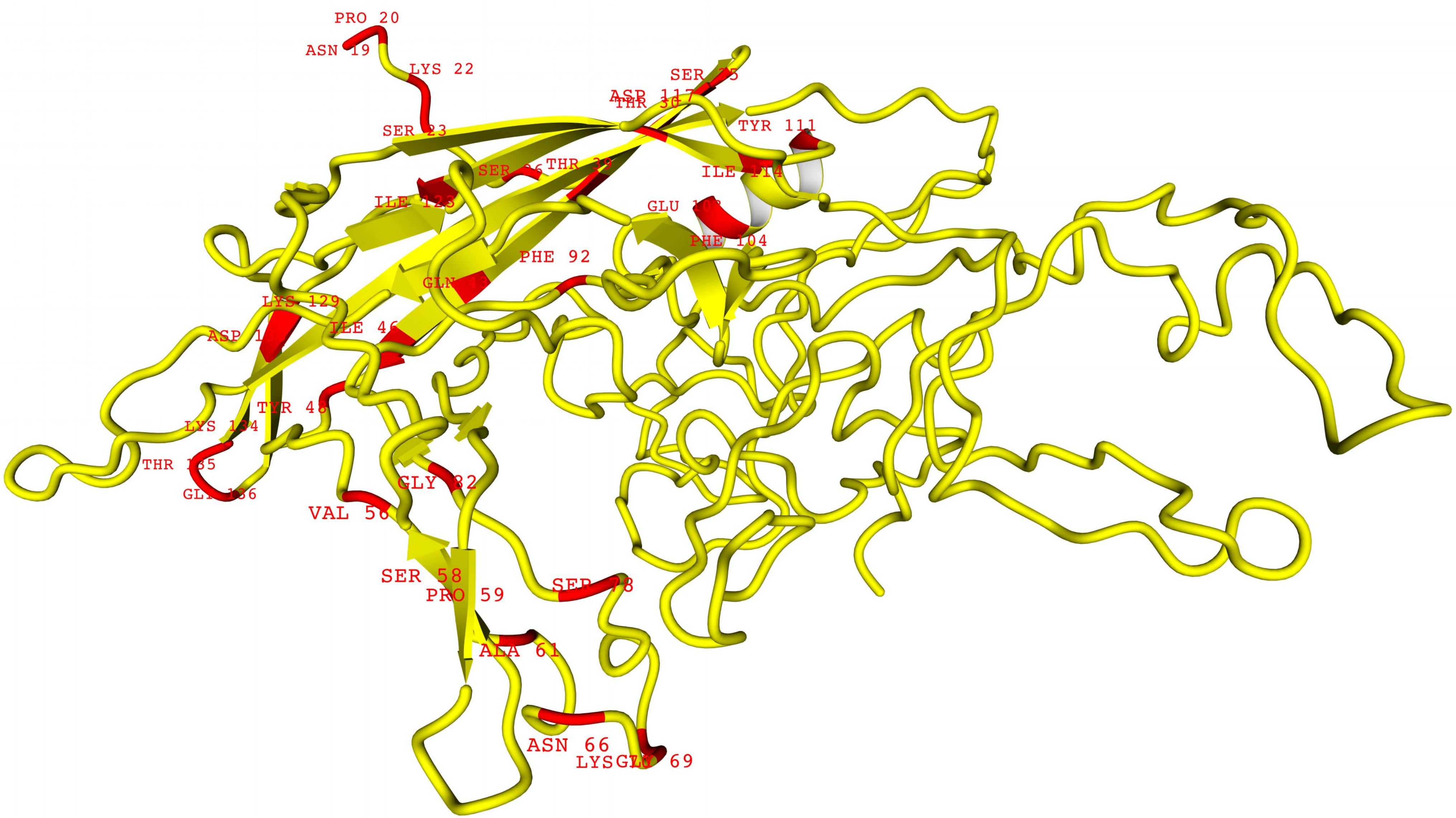

Fig S1. HPV-B19 capsid 3D structure analysis. Exploring amino acid alignments with scores greater than 8.

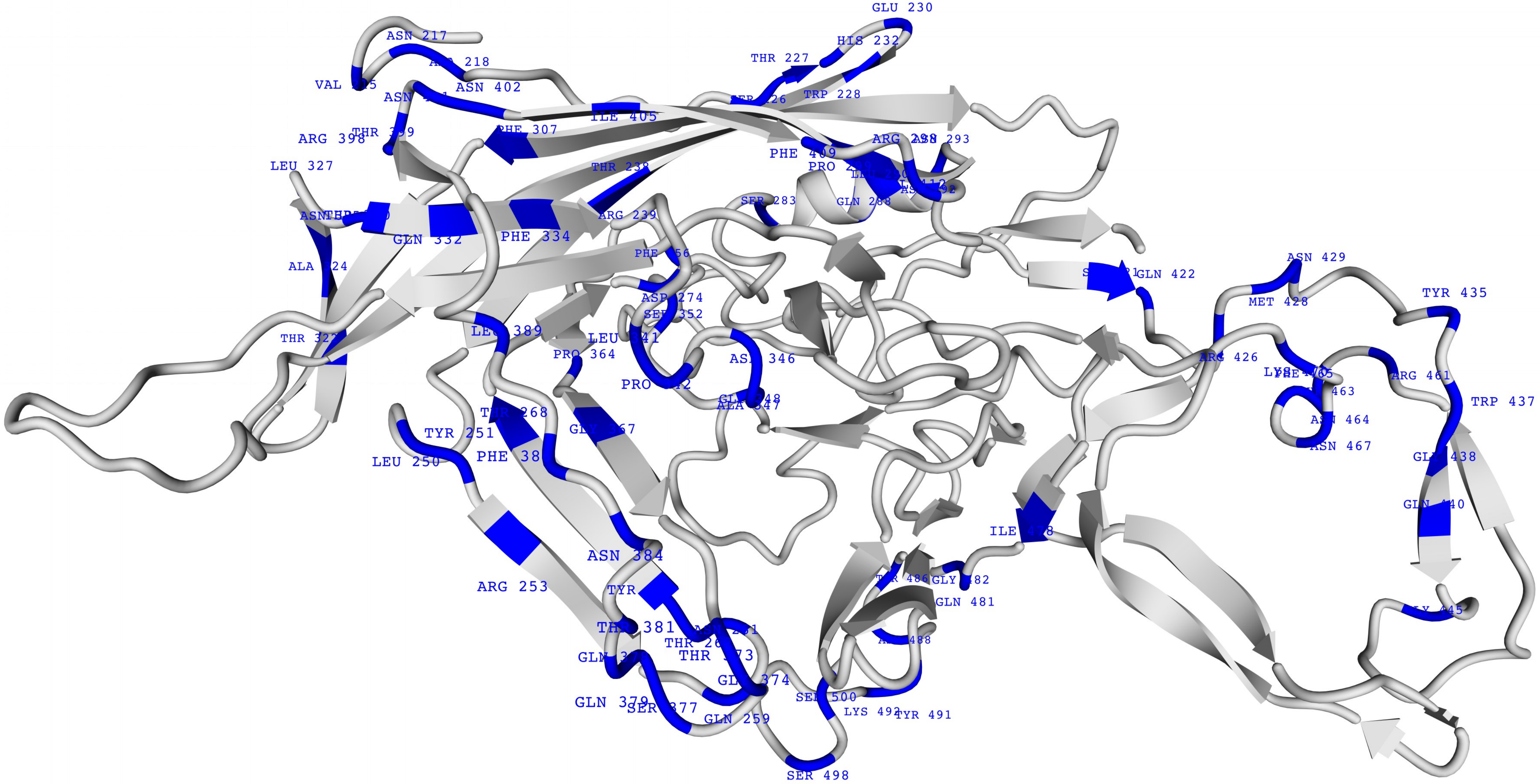

Fig S2. AAV-4 capsid 3D structure assessment. Identification of amino acid alignments with scores exceeding 8.

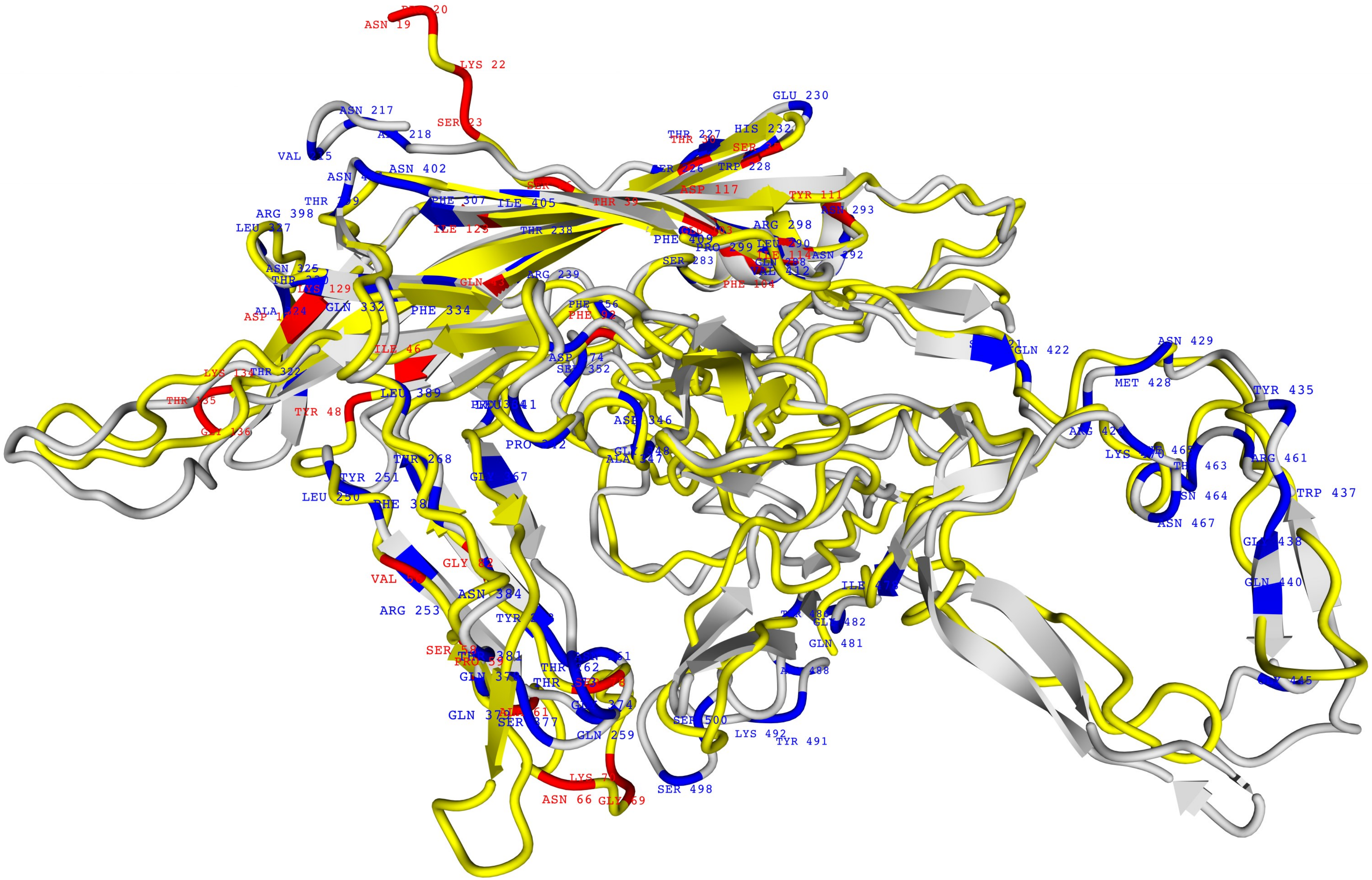

Fig S3. Comparative analysis of viral capsid alignments. Exploring overlapping amino acid alignments in HPV-B19 and AAV-4 capsids.
